# Induction of PGRN by influenza virus inhibits the antiviral immune responses through downregulation of type I interferons signaling

**DOI:** 10.1371/journal.ppat.1008062

**Published:** 2019-10-04

**Authors:** Fanhua Wei, Zhimin Jiang, Honglei Sun, Juan Pu, Yipeng Sun, Mingyang Wang, Qi Tong, Yuhai Bi, Xiaojing Ma, George Fu Gao, Jinhua Liu

**Affiliations:** 1 Key Laboratory of Animal Epidemiology and Zoonosis, Ministry of Agriculture, College of Veterinary Medicine and State Key Laboratory of Agrobiotechnology, China Agricultural University, Beijing, China; 2 College of Agriculture, Ningxia University, Yinchuan, China; 3 CAS Key Laboratory of Pathogenic Microbiology and Immunology, Collaborative Innovation Center for Diagnosis and Treatment of Infectious Disease, Institute of Microbiology, Center for Influenza Research and Early-Warning (CASCIRE), Chinese Academy of Sciences, Beijing, China; 4 State Key Laboratory of Microbial Metabolism, Sheng Yushou Center of Cell Biology and Immunology, School of Life Science and Biotechnology, Shanghai Jiao Tong University, Shanghai, China; 5 Department of Microbiology and Immunology, Weill Cornell Medical College, New York, New York, United States of America; University of North Carolina at Chapel Hill, UNITED STATES

## Abstract

Type I interferons (IFNs) play a critical role in host defense against influenza virus infection, and the mechanism of influenza virus to evade type I IFNs responses remains to be fully understood. Here, we found that progranulin (PGRN) was significantly increased both *in vitro* and *in vivo* during influenza virus infection. Using a PGRN knockdown assay and PGRN-deficient mice model, we demonstrated that influenza virus-inducing PGRN negatively regulated type I IFNs production by inhibiting the activation of NF-κB and IRF3 signaling. Furthermore, we showed that PGRN directly interacted with NF-κB essential modulator (NEMO) via its Grn CDE domains. We also verified that PGRN recruited A20 to deubiquitinate K63-linked polyubiquitin chains on NEMO at K264. In addition, we found that macrophage played a major source of PGRN during influenza virus infection, and PGRN neutralizing antibodies could protect against influenza virus-induced lethality in mice. Our data identify a PGRN-mediated IFN evasion pathway exploited by influenza virus with implication in antiviral applications. These findings also provide insights into the functions and crosstalk of PGRN in innate immunity.

## Author summary

The innate immune system is the first line of host defense against microbial infection, while viruses develop several strategies to evade the host defense. It is of great significance to explore the mechanism by which viruses to evade the antiviral host defense. Previous studies have found that progranulin (PGRN) plays an important role in a variety of physiologic and disease processes. Here, we demonstrated that PGRN induced by influenza virus negatively regulated type I IFN production by inhibiting the activation of NF-κB and IRF3 signaling. We further showed that PGRN directly interacted with NEMO via its Grn CDE domains and recruited A20 to deubiquitinate K63-linked polyubiquitin chains on NEMO. Macrophage played a major source of PGRN during influenza virus infection, and PGRN neutralizing antibodies could protect against influenza virus-induced lethality in mice. Our findings highlight a new strategy whereby influenza virus to evade type I IFN-mediated antiviral immune response and also provide insights into the functions and crosstalk of PGRN in innate immunity.

## Introduction

Influenza virus is one of the most important causes of respiratory tract infection, resulting in approximately 290,000–650,000 deaths each year worldwide (http://www.who.int/news-room/fact-sheets/detail/influenza). Influenza pandemics occur when a novel virus emerges against which a majority of the population has little or no immunity. At least four well-documented influenza pandemics have occurred during the 20^th^ century: the 1918 Spanish pandemic, the 1957 H2N2 pandemic, the 1968 H3N2 Hong Kong pandemic, and the 2009 H1N1 pandemic[1]. Influenza viruses continue to evolve, and new antigenic variants emerge annually, giving rise to seasonal outbreaks. Currently, pandemic influenza A (H1N1) 2009 virus and influenza A (H3N2) virus are the circulating seasonal influenza A virus (IAV) subtypes. Moreover, avian influenza viruses pose a growing threat to human health, especially the H5, H7 and H9 subtypes prevalent in poultry. To date, at least 1,623 human cases (623 deaths) of H7N9 infection[2] and 860 human cases (454 deaths) of H5N1 infection have been reported by the WHO (http://www.who.int/influenza/human_animal_interface). Although avian H9N2 viruses have caused comparatively few deaths, H9N2 have been shown to exchange genetic materials with emerging zoonotic influenza viruses such as H7N9 and H10N8 subtypes[3, 4]. Challenges related to prediction of future immunogenic epitopes as well as vaccine production and distribution issues often limit vaccine availability. Moreover, use of antiviral drugs has resulted in the widespread emergence of influenza strains that are resistant to antiviral drugs, such as adamantanes and neuraminidase inhibitors. Therefore, the development of effective interventions against influenza virus infection remains an urgent public health need. A promising strategy is to identify novel host factors crucial for viral infection, to understand their interplay with influenza viruses, and subsequently to manipulate them to strengthen host defense against the virus.

Progranulin (PGRN) contains 7.5 repeats of a highly-conserved granulin motif[5, 6]. PGRN plays a critical role in a variety of physiologic and disease processes, including inflammatory response[7–10], host defense[11], frontotemporal dementia[12, 13], and lysosomal storage diseases[14]. Brandes *et al*. reveals that PGRN mRNA is induced in the lungs of mice after infection with sublethal or lethal doses of H1N1 virus[15]. Recently, researchers have found that circulating PGRN levels in human patients are correlated with human immunodeficiency virus and hepatitis B virus infection[16, 17]. These findings suggest that PGRN plays a crucial role in viral infection. However, the roles of PGRN in influenza virus infection have not been elucidated.

In this study, we investigated the mechanisms of innate immune responses against influenza virus infection by PGRN. We found that PGRN inhibited the expression of type I IFNs, resulting in increased influenza virus replication in the lung. PGRN-deficient mice were protected against influenza virus infection with much more type I IFN production. We further demonstrated that PGRN inhibited NF-κB and IRF3 activation via recruitment of A20 and deubiquitination of NEMO (also known as IKKγ). Furthermore, we found that macrophage played a major source of PGRN during influenza virus infection, and PGRN neutralizing antibodies could protect against influenza virus-induced lethality in mice.

## Materials and methods

### Ethics statement

All animal studies were performed in accordance with institutional guidelines of China Agricultural University (CAU) (approval SKLAB-B-2010-003) and approved by the Beijing Association for Science and Technology of China (approval SYXK, Beijing, 2007–0023). The use of sera from 6 H7N9-infected patients and 6 healthy volunteers were approved by the review board of the Chinese Center for Disease Control and Prevention (China CDC). Sera were collected by the China CDC after informed consent given was written.

### Mice

PGRN-deficient (PGRN KO) mice were kindly provided by Dr. Wei Tang (Shandong University School of Medicine) and Dr. Xiaojing Ma (Department of Microbiology and Immunology, Weill Cornell Medical College).

### Viruses and cells

Influenza A/Puerto Rico/8/1934 (PR8; H1N1), A/duck/Shandong/F0501-191 /2017 (H5N6), A/chicken/Hebei/LC/2008 (H9N2), and A/Anhui/1/2005 (H5N1) viruses were maintained in our lab. Viruses were propagated in 10-day-old embryonated eggs or Madin-Darby canine kidney cells (MDCK) and titrated to determine the 50% tissue culture infectious dose (TCID_50_) on MDCK cells. MDCK cells, human embryonic kidney (HEK293) cells, and human lung adenocarcinoma epithelial cells (A549) were maintained in DMEM supplemented with 10% (v/v) heat-inactivated fetal bovine serum (FBS; Gibco), 100 U/mL penicillin and 100 μg/mL streptomycin at 37°C under a humidified atmosphere containing 5% CO_2_.

To amplify stocks in MDCKs, cells were infected at an MOI of 0.01 in DMEM, 10 mM HEPES (Gibco), 0.125% BSA (Gibco), 0.5 μg/mL TPCK trypsin. After 1 h at 37°C, cells were washed and overlaid with infection media. After 48 to 72 hour post-infection (hpi), supernatants were harvested, centrifuged and stored at -80°C.

### Antibodies

Anti-progranulin (ab191211), anti-TBK1 (ab40676) and anti-IKKγ (NEMO) (ab178872) antibodies were purchased from Abcam. Rabbit anti-p65 (#10745-1-AP) and anti-IκB (#10268-1-AP) antibodies were from Proteintech. Anti-IRF3 (YT2398) and anti-IKKα/β (YT2302) antibodies were from ImmunoWay Biotechnology Company. Anti-Phospho-TBK1 (Ser172; #5483), anti-Phospho-IRF3 (Ser396; #4947), anti-Phospho-IKKα/β (Ser176/180; #2697), anti-FLAG (#8146), anti-Myc (#2278), and anti-Phospho-p65 (Ser536; #3033) antibodies were purchased from Cell Signaling Technology.

### Clinical specimens

Laboratory confirmation of H7N9 virus infection was performed according to protocols described previously[18, 19].

### Isolation of bone marrow-derived macrophages (BMDMs)

Murine BMDMs were isolated from aseptically dissected and flushed tibias and femurs of 7- to 8-week-old mice. Bone marrow cells were differentiated into BMDMs for 7 days in RPMI-1640 medium supplemented with 10% FBS, 2 mM L-glutamine, 1 mM sodium pyruvate, 1% essential and nonessential amino acids, 100 U/mL each of penicillin and streptomycin, and 100 ng/mL recombinant macrophage colony-stimulating factor. Macrophages were replated 24 h before the experiment.

### Virus infection *in vivo*

6- to 8-week-old wild-type (WT) and PRGN KO mice were anesthetized and infected intranasally with a higher dose (1×10^4^ TCID_50_) or a lower dose (1×10^2^ TCID_50_) of PR8 virus in 50 μL of phosphate-buffered saline (PBS) as previously described[20]. Mouse body weight and survival were monitored daily starting at 1 day post-infection (dpi). Bronchoalveolar lavage fluid (BALF) was obtained by washing with 1 mL of PBS and collected after centrifugation, and the concentration of IFN-β in BALF was measured by ELISA.

### Virus titration

TCID_50_ assays were performed on MDCK cells inoculated with 10-fold serially diluted viruses and incubated at 37°C for 72 h. TCID_50_ values were calculated according to the Reed-Muench method.

### Real-time quantitative PCR

Lung tissues from WT and PGRN KO mice were obtained at the indicated time points after PR8 infection. RNA was extracted from homogenized lung tissues using Trizol reagent (Invitrogen) and cDNA was generated from 1 μg of total RNA using Superscript III First-Strand Synthesis SuperMix (Invitrogen) according to the manufacturer’s protocol. Real-time PCR was conducted using 2× SYBR green PCR master mix (Applied Biosystem). Expression values were normalized to expression of GAPDH and quantified by the 2^−ΔΔ*CT*^ method. The gene-specific primers used were listed in S1 Table.

### siRNA-mediated gene silencing

To knock down the indicated target genes, chemically synthesized siRNAs as well as negative control (NC) siRNA were obtained from GenePharma Company. Cells were transfected with 50 nM siRNA in 2.0 μL Lipofectamine RNAiMAX (Invitrogen) for approximately 36 h, and then used for the subsequent analyses.

### Western blotting

Cells were lysed in radio-immunoprecipitation assay (RIPA) buffer containing 1 mM phenylmethylsulfonyl fluoride and the total protein content was measured with a bicinchoninic acid protein assay kit (Beyotime, China). Similar amount of sample was separated on a 12% sodium dodecyl sulfate (SDS)-polyacrylamide gel, and then electroblotted onto a polyvinylidene difluoride (PVDF) membrane. After blocking in Tris-buffered saline (10 mM Tris-HCl, pH 8.0, containing 150 mM NaCl) containing 5% (w/v) non-fat dry milk and 0.5% (v/v) Tween-20, membranes were incubated with primary antibodies for 2 h. After washing, the appropriate secondary antibody (horseradish peroxidase-conjugated species-specific antisera; 1:5000 dilution) was added and incubated for 1 h. Bound antibody was visualized using an enhanced chemiluminescence system (Thermo Fisher). The expression of cytosolic proteins was normalized to β-actin.

### Histology

WT and PGRN KO mice were euthanized and sacrificed. The lungs were fixed with 4% formaldehyde. After fixation and processing in paraffin wax, sections (5 mm thick) were cut longitudinally through the left and right lung and stained with hematoxylin and eosin (H&E) for assessment of general histopathology.

### Immunohistochemistry

The lung sections were deparaffinized in xylene, rehydrated in grade alcohols and washed in distilled water. To block endogenous peroxidase activity, slides were incubated with 3% H_2_O_2_ in methanol. The lung sections were stained with anti-PGRN antibody (1:100 dilution; ab191211) at 4°C overnight in a humidified chamber, then incubated with horseradish peroxidase-conjugated secondary antibody for 60 min at room temperature. Signal was detected using the Vector Elite ABC Kit (Vectastain, Vector). The tissue sections were also stained with H&E for routine morphologic analysis.

### Luciferase assay

HEK293 cells were transfected with: (i) 150 ng of NF-κB-luciferase or IFN-β-luciferase reporter plasmid; (ii) 0, 100 or 250 ng of a vector encoding PGRN; (iii) 0, 150 or 250 ng of pcDNA3.1; and (iv) 250 ng of a vector encoding NF-κB signaling molecules (MyD88, TRAF6, IKKβ, NEMO, p65) and IFN-β signaling molecules (RIG-I, MAVS, TBK1, NEMO, IRF3) using the jetPRIME kit (Polyplus Transfection). At 24 h after transfection, cells were lysed and luciferase activity was analyzed using the Dual-Luciferase Reporter Assay System (Promega) according to the manufacturer’s protocol. Data were normalized for transfection efficiency by dividing firefly luciferase activity by renilla luciferase activity.

### Co-immunoprecipitation (Co-IP)

HEK293 cells were transfected with 2 μg of a vector-encoding FLAG-PGRN and 2 μg of a vector encoding myc-NEMO using the jetPRIME kit (Polyplus Transfection). After 24 h transfection, cells were washed with PBS and lysed in 250 μL of RIPA buffer containing protease and phosphatase inhibitors (Roche). Protein lysates (500 μg) were used for Co-IP. Lysates were incubated with anti-FLAG or anti-HA antibodies overnight at 4°C and then protein A/G agarose beads (Santa Cruz) were added to the samples for 1–1.5 h at 4°C. The beads were washed with lysis buffer, and analyzed by western blotting.

### Confocal microscopy

HEK293 cells were co-transfected with expression plasmids encoding FLAG-tagged PGRN and Myc-tagged NEMO. After 24 h transfection, cells were infected with PR8 virus at an MOI of 2 in serum-free medium for 6 h. Cells were washed with 0.01 M PBS and fixed in 4% PFA for 15 min, permeabilized with 0.2% Triton X-100, and then blocked for 60 min at room temperature with PBS containing 2% bovine serum albumin and 7% FBS. Cells were incubated overnight at 4°C with primary antibodies, then with Alexa Fluor 555-conjugated goat anti-mouse IgG and Alexa Fluor 488-conjugated goat anti-rabbit IgG for 1 h. Finally, cover slips were mounted onto microscope slides with 10–20 μL of DAPI for 3 min and examined by confocal microscopy. Images were processed using FluoView FV1200 confocal laser scanning microscope (Olympus) and analyzed by the Imaris 9.2 platform.

### Ubiquitination assay

HEK293 cells were transfected with expression plasmids encoding Myc-NEMO with or without co-expression of FLAG-tagged PGRN or PGRN mutants and HA-Ubiquitin or HA-Ubiquitin mutants (K48 or K63). After 24 h transfection, cells were harvested and lysed in RIPA buffer (50 mM Tris-HCl (pH 8.0), 150 mM NaCl, 1% NP-40, 0.1% SDS, and 1 mM EDTA) containing protease inhibitor cocktail and 10 μM deubiquitinase inhibitor *N*-ethylmaleimide (NEM, Sigma). The cell extracts were immunoprecipitated with anti-Myc antibody overnight at 4°C and then beads were added to the samples for 1–1.5 h at 4°C. The beads were washed three times with RIPA buffer and analyzed by immunoblotting with an anti-HA antibody.

### *In vivo* depletion of macrophage

Six-week-old male WT and KO mice were injected with 100 μL of Clodronate Liposomes (CL, Sigma, St. Louis, MO) or PBS containing liposomes two times via the intranasal route, at 2 days before influenza virus infection and day 2 after PR8 virus infection. The survival rate was measured for the whole experiment.

### *In vivo* protection studies in mice

PGRN polyclonal antibodies (R&D systems) and polyclonal goat IgG were used for PGRN neutralization as described[21]. Six-week-old male C57BL/6 mice were passively administered 200 μg of IgG control or PGRN polyclonal antibodies via intraperitoneal injection 1 day prior to inoculation with PR8 virus at a dose of 1×10^4^ TCID_50_ via the intranasal route. Animals were monitored daily for morbidity.

### Flow cytometry

Lungs were excised and digested enzymatically at 37°C for 30 min in PBS with 5% FBS, 3 mg/ml collagenase type IV (Worthington), and 20 U/ml DNase (Roche). The digested tissues were then filtered through 70 μm nylon filters (BD Biosciences), and then cells were washed with sterile HBSS. Following RBC lysis, single cell suspensions were stained with CD11b-APC-cy7, CD11c-PE and Ly6G-FITC antibodies. The samples were acquired on MACSQuant VYB flow cytometer (Miltenyi Biotech) and data were analyzed using FlowJo Software (Ashland, OR). Cell sorting was performed on BD Influx Cell Sorting System.

### Statistical analysis

All experiments were repeated at least three times with consistent results. All statistical analyses were performed using GraphPad Prism software version 5.00 (GraphPad Software Inc., San Diego, CA, USA). Differences among experimental groups were assessed using analysis of variance (ANOVA). Kaplan-Meier method was employed for survival analysis. *p* values less than 0.05 were considered statistically significant.

## Results

### PGRN modulates influenza virus infection *in vitro* and *in vivo*

**Fig 1:**
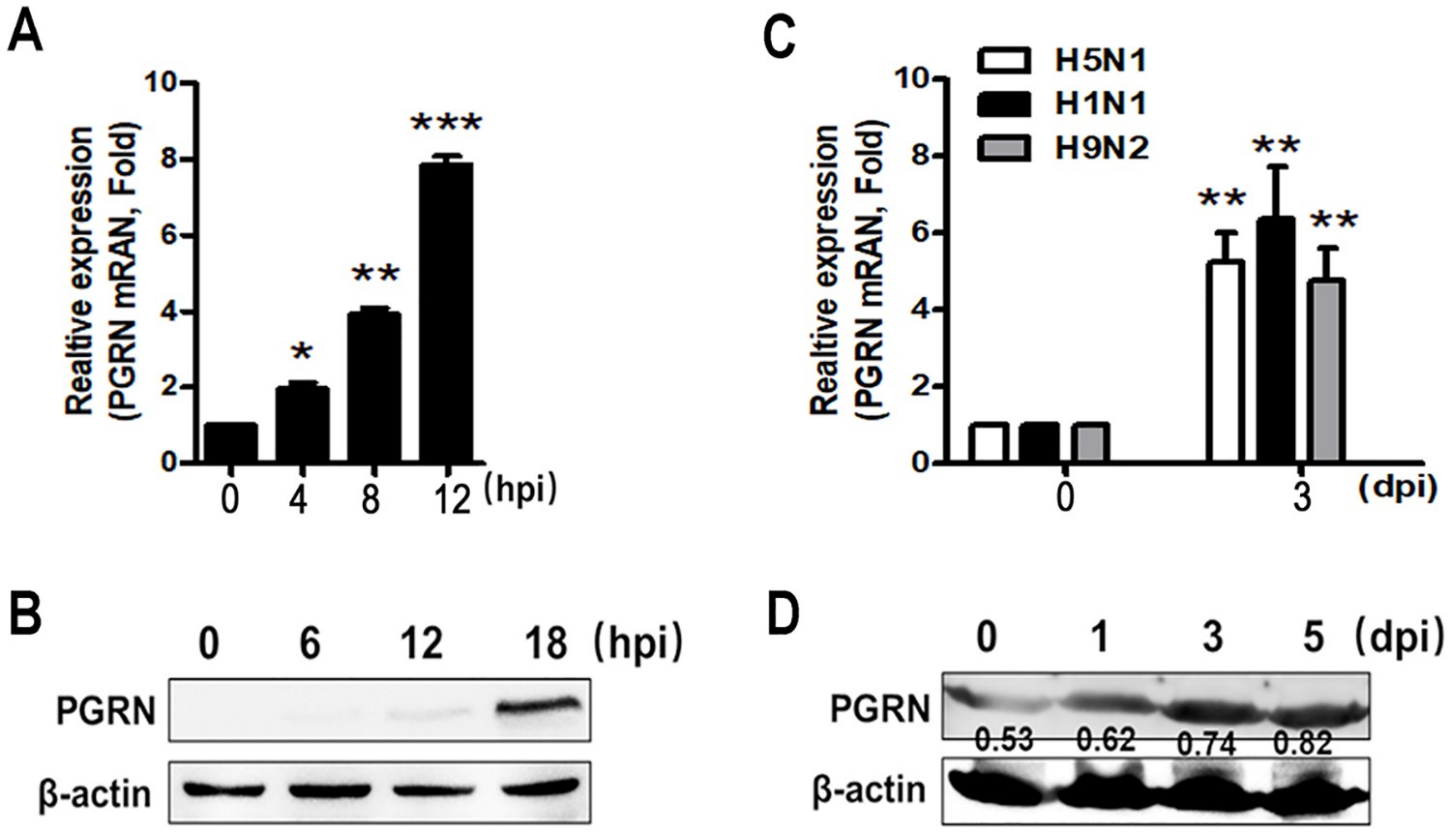
PGRN is induced during influenza virus infection. (A) PGRN mRNA expression in A549 cells infected with live PR8 virus at an MOI of 1 at 4, 8 and 12 hpi was analyzed by q-PCR. (B) PGRN protein level in A549 cells infected with live PR8 virus at an MOI of 1 at 6, 12 and 18 hpi was examined by western blot. β-actin is shown as a loading control. (C) PGRN mRNA expression in the lung homogenates of mice challenged with H5N1 (1×10^2^ TCID_50_), PR8 (1×10^3^ TCID_50_) or H9N2 (1×10^4^ TCID_50_) viruses at 3 dpi was determined by q-PCR. (D) PGRN protein level in the lung tissue homogenates of mice challenged with PR8 (1×10^3^ TCID_50_) virus at the indicated time points was evaluated by western blot. (A) and (C): Each data is represented as means ± SEMs and is representative of three independent experiments. *p < 0.05; **p < 0.01; ***p < 0.001. (B) and (D): Data are repeated three times and the representative results are shown.

To assess whether PGRN was involved in influenza infection *in vitro*, we infected A549 cells with PR8 virus and then analyzed PGRN expression. As shown in Fig 1A, PGRN mRNA expression was significantly elevated in PR8 virus-infected A549 cells at 4 (*p* < 0.05), 8 (*p* < 0.01) and 12 (*p* < 0.001) hpi. Western blot results demonstrated that PR8 virus evidently upregulated the expression of PGRN at 12 and 18 hpi (Fig 1B). To further determine the expression profiles of PGRN in influenza infection *in vivo*, we infected mice with live H5N1, PR8 or H9N2 viruses. We found that H5N1, PR8 or H9N2 viruses significantly induced PGRN mRNA expression in the lung tissue homogenates of mice at 3 dpi (Fig 1C) (*p* < 0.01). PR8 virus also upregulated the protein level of PGRN in the lung tissue homogenates of mice at 1, 3 and 5 dpi (Fig 1D). PGRN level in the BALF (D3, *p* = 0.0117; D5, *p* = 0.0038; D7, *p* = 0.0182; D9, *p* = 0.0068) (S1A Fig) and serum (D3, *p* = 0.0177; D5, *p* = 0.0055; D7, *p* = 0.0041; D9, *p* = 0.0040) (S1B Fig) in mice was rapidly elevated starting at 3 dpi. To quantitate PGRN level in human with influenza infection, we collected serum samples from 6 H7N9-infected patients during the acute phase of infection. Our results showed that PGRN was significantly increased in H7N9 virus-infected patients compared to healthy controls (*p* = 0.0006) (S1C Fig). Increased PGRN in lung tissues was further validated by immunohistochemistry assays after H5N1, PR8 or H9N2 viruses infection. The results showed that H5N1, PR8 or H9N2 viruses infection clearly induced the upregulation of PGRN at 3, 5 and 7 dpi (S1D Fig). Stronger PGRN staining was also observed in areas of inflammatory cell infiltration at 5 and 7 dpi (S1D Fig).

To determine if the induction of PGRN in infected cells is dependent on virus replication, we inoculated A549 cells with live H1N1, H9N2, UV-inactivated H1N1, or UV-inactivated H9N2 virus particles. Our results showed that the induction of PGRN by flu virus was dependent on virus replication (S1E Fig). Furthermore, we transfected HEK293 cells with PR8 protein-coding plasmids, and then analyzed the PGRN expression. We found that the viral internal protein NS1, PB1, and PB2 induced PGRN, respectively (S1F Fig).

**Fig 2:**
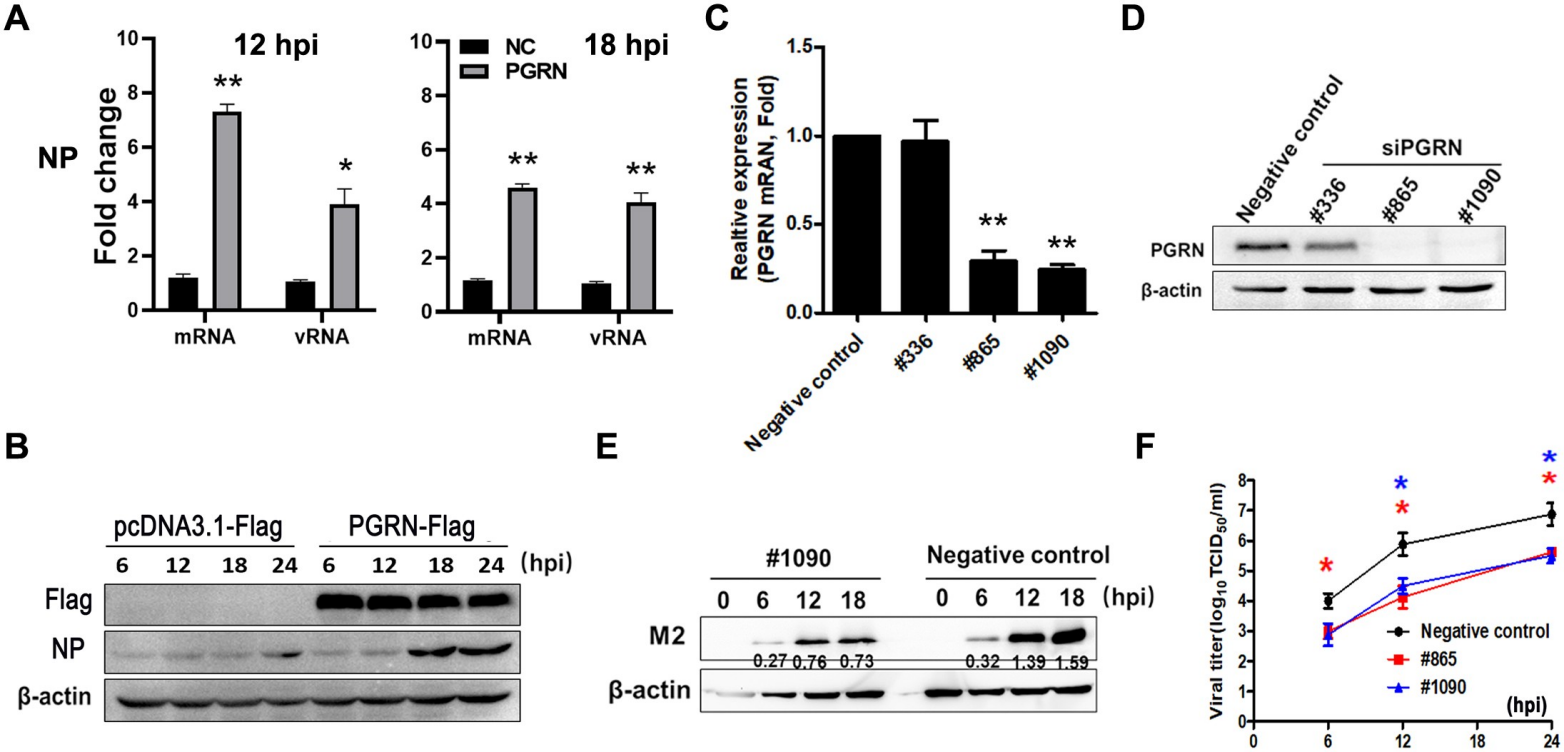
PGRN plays a functional role during influenza virus replication. (A) A549 cells were transfected with negative control (NC) or PGRN-expressing plasmids, and 24 h after transfection, cells were infected with live PR8 virus at an MOI of 1. The mRNA and vRNA of NP was analyzed by q-PCR at 12 and 18 hpi. Data are representative of three independent experiments. *p < 0.05; **p < 0.01; ***p < 0.001. (B) A549 cells were transfected with pcDNA3.1-Flag or PGRN-Flag plasmids, and 24 h after transfection, cells were infected with live PR8 virus at an MOI of 1. The NP protein in A549 cells was analyzed by western blot at 6, 12, 18 and 24 hpi. β-actin is shown as a loading control. Data are representative of three independent experiments. (C) Serum-starved A549 cells were transfected with siRNAs (either negative controls or PGRN-targeting siRNAs #336, #865 and #1090) and PGRN mRNA expression was measured 24 h later by q-PCR. Each data is representative of three independent experiments. **p < 0.01 (D) Serum-starved A549 cells were transfected with siRNAs (either negative controls or PGRN-targeting siRNAs #336, #865 and #1090) and PGRN protein level was examined 48 h later by western blot. Data are representative of three independent experiments. (E) A549 cells were transfected with siRNAs (either negative control or PGRN-targeting siRNA #865 or #1090) after PR8 infection at an MOI of 1. Viral titers were measured by TCID_50_ assay at the indicated time points. Each data is represented as means ± SEMs and is representative of three independent experiments. *p<0.05; **p<0.01. (F) Serum-starved A549 cells were transfected with negative control or PGRN-targeting siRNA #1090. After 48 h, the cells were infected with PR8 virus at an MOI of 1 and expression of M2 protein was measured by western blot. Data are representative of three independent experiments.

To assess whether PGRN affects viral replication, we transfected A549 cells with PGRN-expressing plasmids. At 24 h post transfection, A549 cells were infected with live PR8 virus at an MOI of 1. We found that over-expression of PGRN enhanced mRNA and vRNA of NP in A549 cells at 12 and 18 hpi (Fig 2A) and NP level in A549 cells after PR8 infection (Fig 2B). Furthermore, we used siRNA to silence PGRN and then infected cells with PR8 virus at an MOI of 1. We found that transfection of cells with PGRN-targeting siRNA#865 and #1090 significantly decreased the mRNA expression (Fig 2C) and protein level of PGRN (Fig 2D). Furthermore, transfection of siRNA#865 and #1090 in A549 cells significantly decreased virus titers at 12 and 24 hpi (Fig 2F) (*p* < 0.05). Transfection of siRNA#1090 in A549 cells inhibited the expression of viral M2 protein with a time-dependent manner at 12 and 18 hpi (Fig 2E). Overall, these data suggest that PGRN is involved in influenza virus infection.

### PGRN KO mice are resistant to influenza virus infection

**Fig 3:**
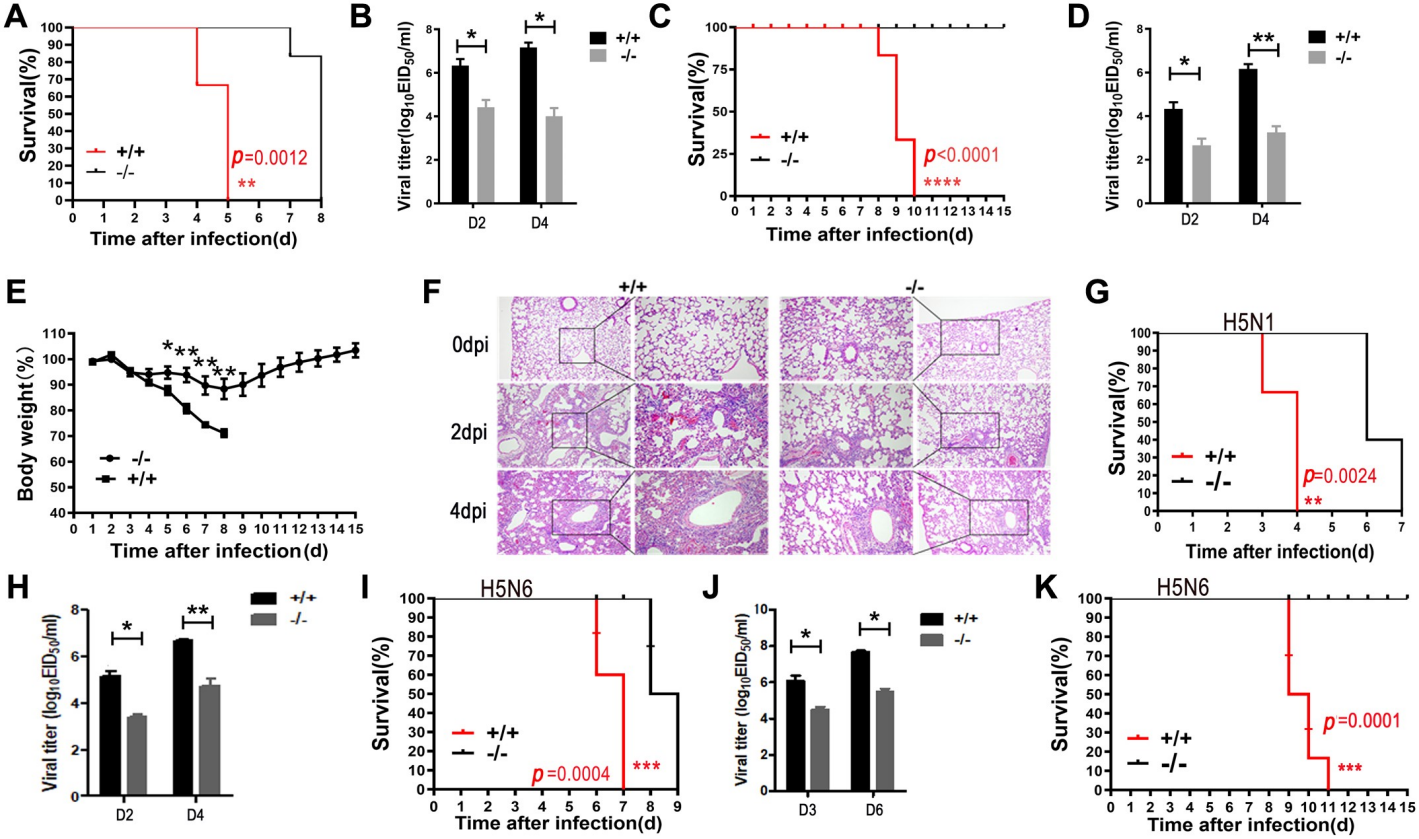
PGRN-deficient mice are resistant to influenza virus infection. (A) Survival of WT and KO mice after infection with 1×10^4^ TCID_50_ of PR8 virus. (B) Viral titers in the lungs of WT and KO mice on days 2 and 4 after infection with PR8 virus (1×10^4^ TCID_50_) were determined by EID_50_ assay. Data are from three independent experiments with n = 6 mice per group run in triplicate. Error bars indicate SEM. *p < 0.05; **p < 0.01. (C) Survival of WT and KO mice after infection with 1×10^2^ TCID_50_ of PR8 virus. (D) Viral titers in the lungs of WT and KO mice on days 2 and 4 after PR8 infection (1×10^2^ TCID_50_) were determined by EID_50_ assay. Data are from three independent experiments with n = 6 mice per group run in triplicate. Error bars indicate SEM. *p < 0.05; **p < 0.01. (E) WT and KO mice (n = 6 per genotype) were infected with 1×10^2^ TCID_50_ of PR8 virus. Changes in body weight were monitored daily. Each data point represents as the means ± SEMs and is representative of three independent experiments. *p < 0.05; **p < 0.01. (F) H&E staining of lung tissues from WT and KO mice after challenge with 1×10^4^ TCID_50_ of PR8 virus. Representative of H&E staining images from 6 mice per group of three independent experiments. (G) Survival of WT and KO mice after infection with 1×10^4^ TCID_50_ of H5N1 virus. (H) Viral titers in the lungs of WT and KO mice on days 2 and 4 after infection with H5N1 virus (1×10^4^ TCID_50_) were determined by EID_50_ assay. Data are from three independent experiments with n = 6 mice per group run in triplicate. Error bars indicate SEM. *p < 0.05; **p < 0.01. (I) Survival of WT and KO mice after infection with 1×10^4^ TCID_50_ of H5N6 virus. (J) Viral titers in the lungs of WT and KO mice on days 3 and 6 after infection with H5N1 virus (1×10^4^ TCID_50_) were determined by EID_50_ assay. Data are from three independent experiments with n = 6 mice per group performed in triplicate. Error bars indicate SEM. *p < 0.05. (K) Survival of WT and KO mice infected with 1×10^2^ TCID_50_ of H5N6 virus. (A), (C), (G), (I) and (K): Data are pooled from three independent experiments with n = 10 mice per group. Kaplan-Meier Survival Curves are compared using the log-rank (Mantel-Cox) analysis. *p < 0.05; **p < 0.01, ***p < 0.001.

To further investigate the functional significance of PGRN in influenza virus infection, we intranasally infected PGRN KO mice with PR8 virus at a higher dose of 1×10^4^ TCID_50_ and monitored the survival of the animals. Prior to the experiment, we confirmed the genotypes of WT and KO mice by PCR (S2 Fig). All of the WT mice died by 5 dpi, whereas nearly all KO mice died by 8 dpi (Fig 3A). Using an 50% egg infective doses (EID_50_) assay, we found that PR8 viral loads in lung homogenates of KO mice were significantly lower than those of WT mice on 2 (*p* < 0.05) and 4 (*p* < 0.01) dpi (Fig 3B), indicating that PGRN deficiency results in reduced viral replication. We also infected WT and KO mice with PR8 virus at a lower dose of 1×10^2^ TCID_50_. Interestingly, no KO mice died, while mortality of WT mice reached 100% by 10 dpi (Fig 3C). Viral titers in lung homogenates of KO mice were significantly lower than those of WT mice on 2 (*p* < 0.05) and 4 (*p* < 0.01) dpi (Fig 3D). Both WT and KO mice began to lose weight on 3 dpi and reached maximum weight loss on 8 dpi (Fig 3E). However, KO mice suffered significantly less weight loss than WT mice and started to regain body weight by 4 or 5 dpi, while WT mice continued to lose body weight until death (Fig 3E). Moreover, histopathology results revealed that KO mice sustained a lesser degree of lung injury, including edema, alveolar hemorrhaging, alveolar wall thickening, and inflammatory cell infiltration, compared with WT mice (Fig 3F).

To determine whether the resistance of KO mice to influenza virus infection was strain-specific, we challenged WT and KO mice with highly pathogenic avian influenza viruses of the H5N1 and H5N6 subtypes. After H5N1 virus infection at a dose of 1×10^4^ TCID_50_, all WT mice died by 4 dpi, whereas KO mice died by 7 dpi (Fig 3G). Viral loads in the lungs of KO mice were significantly lower than those in lungs of WT mice on 2 (*p* < 0.05) and 4 (*p* < 0.01) dpi (Fig 3H). In a separate experiment, we infected mice with H5N6 virus at a higher dose of 1×10^4^ TCID_50_. We found that all WT mice died by 7 dpi, whereas KO mice died by 9 dpi (Fig 3I). Viral titers in lung homogenates of KO mice were significantly lower than those of WT mice on 3 (*p* < 0.05) and 6 (*p* < 0.05) dpi (Fig 3J). When infection mice with H5N6 virus at a lower dose of 1×10^2^ TCID_50_, no KO mice died, but 100% mortality was observed in WT mice by 11 dpi (Fig 3K).

Collectively, these results indicate that PGRN is involved in influenza virus replication and the PGRN KO mice are resistant to influenza virus infection.

### PGRN negatively regulates type I IFNs expression upon influenza infection

**Fig 4:**
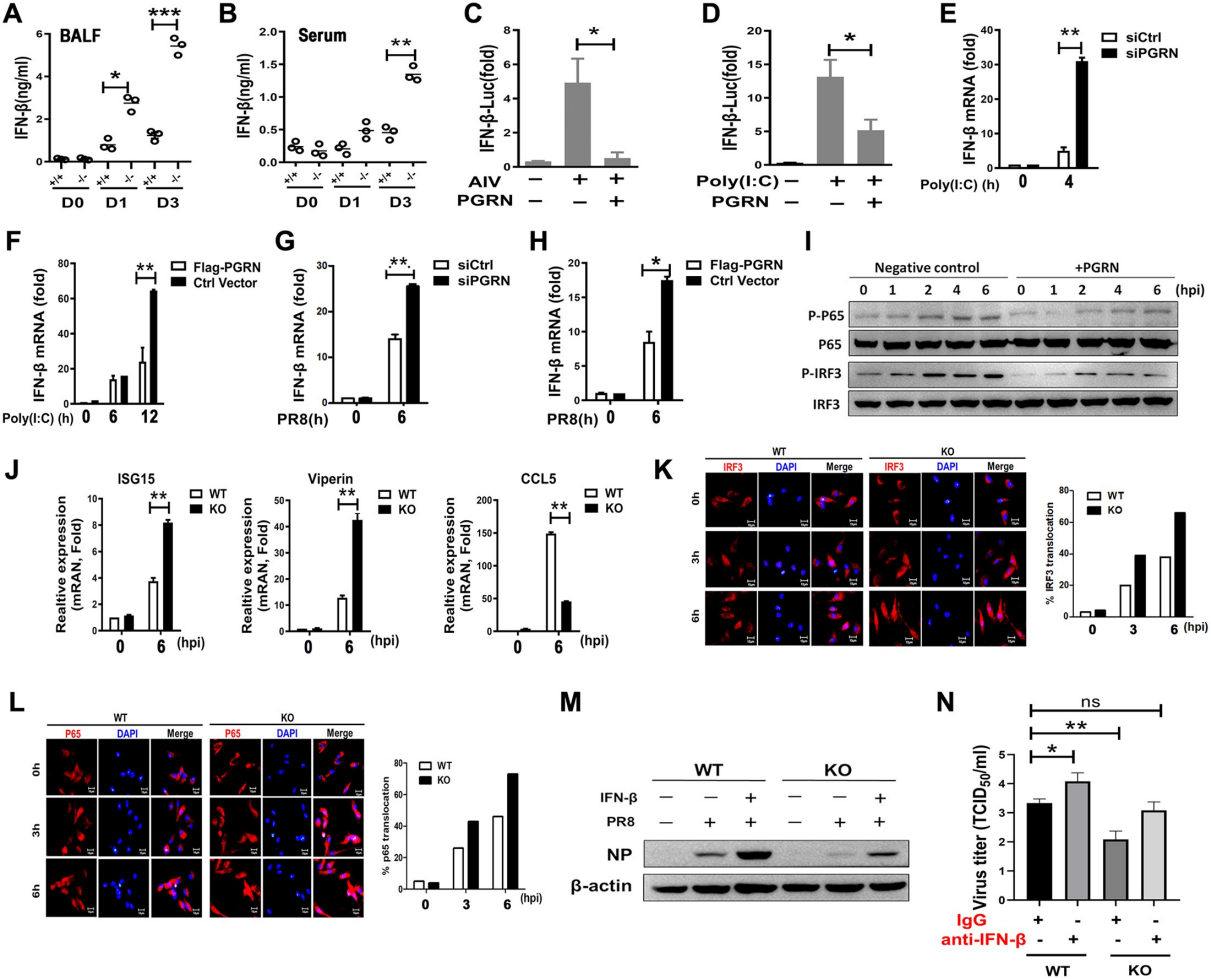
PGRN negatively regulates type I IFN signaling in response to influenza virus infection. (A) IFN-β protein concentrations in BALF on day 1 and 3 after infection with 1×10^4^ TCID_50_ of PR8 virus was determined by ELISA. (B) IFN-β production in serum on day 1 and 3 after infection with 1×10^4^ TCID_50_ of PR8 virus was determined by ELISA. (C) Serum-starved HEK293 cells were transfected with negative control or PGRN-encoding plasmids, and then infected with PR8 virus at an MOI of 1. IFN-β activity was measured using luciferase reporter assays. (D) Serum-starved HEK293 cells were transfected with negative control or PGRN-encoding plasmids, and then transfected with 5 μg/mL of poly(I:C). IFN-β activity was measured using luciferase reporter assays. (E) HEK293 cells were transfected with PGRN-specific siRNA for 36 h, and then transfected with 5 μg/mL of poly(I:C). Expression of IFN-β at the indicated time points was measured by q-PCR. (F) HEK293 cells were transfected with FLAG-PGRN-encoding expression plasmids for 24 h, and then transfected with 5 μg/mL of poly(I:C). Expression of IFN-β at the indicated time points was measured by q-PCR. (G) HEK293 cells were transfected with PGRN-specific siRNA for 36 h, and then infected with PR8 virus at an MOI of 1. Expression of IFN-β at 6 hpi was measured by q-PCR. (H) HEK293 cells were transfected with FLAG-PGRN-encoding expression plasmids for 24 h, and then infected with PR8 virus at an MOI of 1. Expression of IFN-β at 6 hpi was measured by q-PCR. (I) Serum-starved HEK293 cells were transfected with negative control or PGRN- encoding plasmids, and then infected with PR8 virus at an MOI of 1. Activation of p65 and IRF3 at the indicated time points was measured by western blot. Data are representative of three independent experiments. (J) Expression of the IFN-stimulated genes in WT and KO BMDMs infected with PR8 virus was analyzed by q-PCR at 6 hpi. (K) BMDMs from WT or KO mice were infected with PR8 virus at an MOI of 2. The subcellular localization of IRF3 was determined by IRF3 intracellular staining, and confocal fluorescence images were captured. Scale bar represents 10 μm. Representative sections are shown and are representative of three independent experiments. (L) WT or KO BMDMs were infected with PR8 virus at an MOI of 2. The subcellular localization of p65 was determined by p65 intracellular staining, and confocal fluorescence images were captured. Scale bar represents 10 μm. Representative sections are shown and are representative of three independent experiments. (M) WT and KO BMDMs were infected with PR8 virus at MOI of 0.1 in the presence or absence of 25 μg/mL of IFN-β-neutralizing antibody, and NP protein expression was measured by western blot. Data are representative of three independent experiments. (N) Viral titers in WT and KO BMDMs after PR8 infection with an MOI of 0.1 in the presence or absence of 200 μg/mL of IFN-β-neutralizing antibody. (A)-(H), (J) and (N): Data are pooled from three independent experiments performed in triplicate. Error bars indicate SEM. *p<0.05, **p < 0.01, ***p < 0.001.

Type I IFNs play a crucial role in restricting viral replication and enhancing host immune responses against influenza virus infection. Thus, we next evaluated IFN-β levels in BALF and serum from WT and PGRN KO mice after PR8 virus infection. Compared with WT mice, IFN-β levels were significantly increased in the BALF of KO mice on 1 (*p* < 0.05) and 3 (*p* < 0.001) dpi (Fig 4A), as well as in the serum of KO mice on 3 (*p* < 0.01) dpi (Fig 4B). However, PR8 virus- and poly(I:C)-induced IFN-β luciferase activity was remarkably inhibited in PGRN-overexpressing HEK293 cells (*p* < 0.05) (Fig 4C and 4D). Real-time PCR results demonstrated that siRNA silencing of PGRN increased IFN-β mRNA abundance at 4 h after poly(I:C) transfection (*p* < 0.01) (Fig 4E), while overexpression of PGRN resulted in decreased IFN-β expression at 12 h after transfection (*p* < 0.01) (Fig 4F). Furthermore, siRNA silencing of PGRN increased IFN-β mRNA expression at 6 h after PR8 virus infection (*p* < 0.01) (Fig 4G), while overexpression of PGRN resulted in decreased IFN-β expression at 6 hpi (*p* < 0.05) (Fig 4H).

To investigate which factors affect IFN-β expression, HEK293 cells were transfected with PGRN-encoding plasmids, and then infected with PR8 virus at an MOI of 1. Our results indicated that PGRN overexpression remarkably inhibited phospho-p65 level at 4 and 6 hpi (Fig 4I). Moreover, PR8 virus-induced phosphorylation of IRF3 was completely inhibited in PGRN-overexpressing cells (Fig 4I). In addition, significantly increased expression of ISG-15 and viperin and decreased expression of RANTES/CCL5 were observed in BMDMs from KO mice at 6 h after PR8 virus infection (*p* < 0.01) (Fig 4J).

To examine whether PGRN contributes to decreased IRF3 nuclear translocation, p65 and IRF3 localization in WT and KO BMDMs was assessed after PR8 virus infection. We found that significantly increased IRF3 (Fig 4K) and p65 (Fig 4L) nuclear localization was observed in KO BMDMs. The inhibitory effect of PGRN on p65 (S3A Fig) and IRF3 (S3B Fig) nuclear translocation was further verified in PGRN-overexpressing cells.

To address the role of increased type I IFNs during influenza virus infection, we measured viral gene expression and viral titers in BMDMs from WT and PGRN KO mice in the presence of an IFN-β-neutralizing antibody. We found that neutralization of IFN-β led to significantly increased NP expression (Fig 4M) and PR8 virus titers (*p* < 0.05) (Fig 4N) in BMDMs from KO mice, suggesting that the upregulation of IFN-β in KO mice is crucial for the increased resistance to influenza virus infection.

In summary, PGRN deficiency resulted in increased NF-κB and IRF3 activation and type I IFN production, which was associated with decreased viral replication during influenza virus infection. These results suggest that PGRN negatively regulates type I IFN expression during influenza virus infection.

### The inhibition of influenza virus-induced NF-κB and IRF3 activation by PGRN is dependent on NEMO

**Fig 5:**
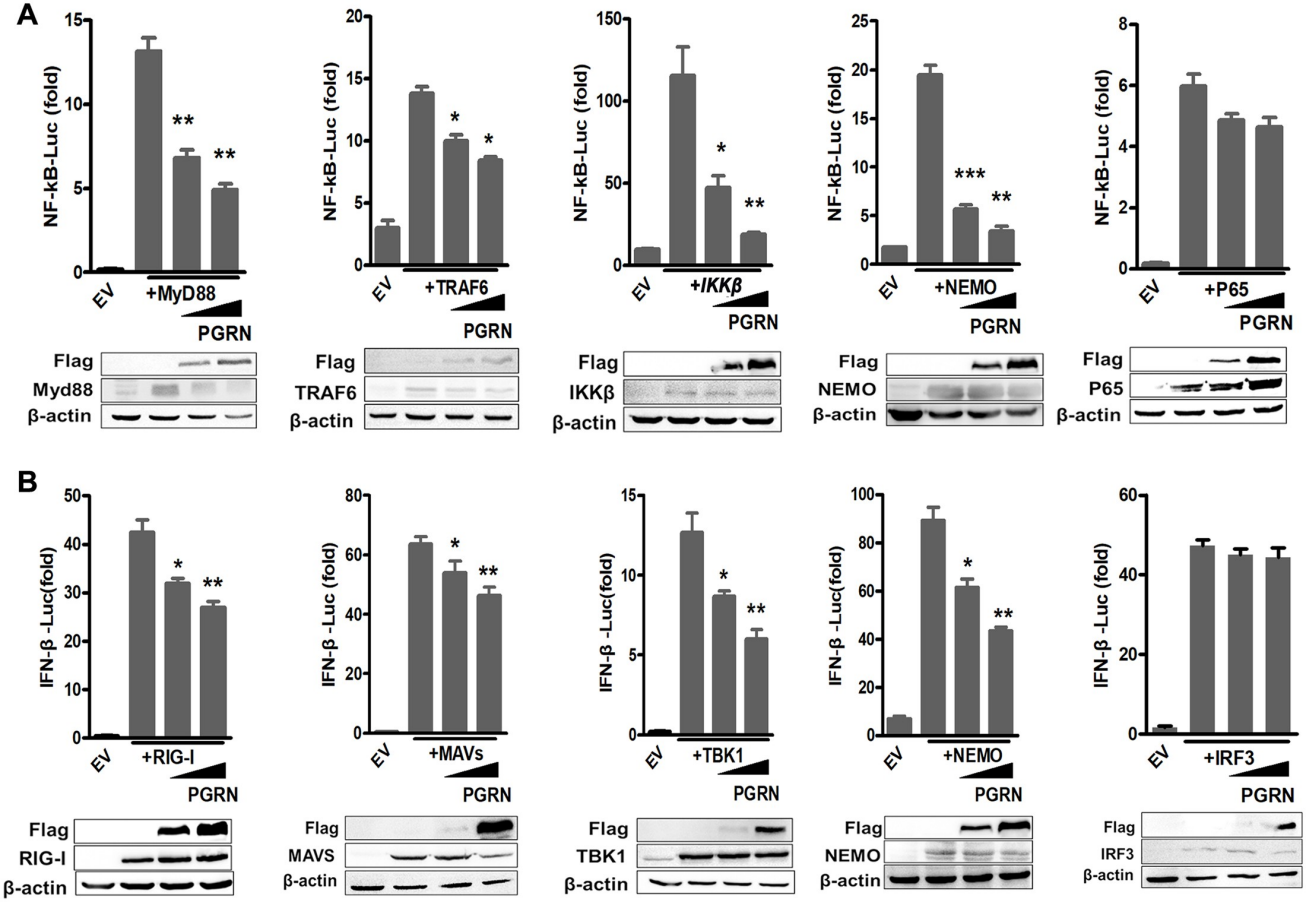
PGRN inhibits influenza virus-induced activation of NEMO. (A) HEK293 cells were transfected with empty vector or plasmids encoding MyD88, TRAF6, IKKβ, NEMO or p65 along with either an empty vector or a PGRN-encoding vector. After 24 h transfection, the overexpressing efficiency of PGRN, MyD88, TRAF6, IKKβ, NEMO or p65 was examined by western blot. And NF-κB activity was measured using luciferase reporter assays. The NF-κB activity data are pooled from three independent experiments performed in triplicate. Error bars indicate SEM. *p<0.05; **p<0.01; ***p<0.001. The western blot results are representative of three independent experiments. (B) HEK293 cells transfected with empty vector or plasmids encoding RIG-I, MAVS, TBK1, NEMO or IRF3 along with either an empty vector or a PGRN-encoding vector. After 24 h transfection, the overexpressing efficiency of PGRN, RIG-I, MAVS, TBK1, NEMO or IRF3 was examined by western blot. IFN-β activity was measured using luciferase reporter assays. The IFN-β activity data are pooled from three independent experiments performed in triplicate. Error bars indicate SEM. *p<0.05; **p<0.01. The western blot results are representative of three independent experiments.

To identify which molecules are involved in PGRN modulation of the type I IFN signaling pathway, we examined the sequence pattern recognition receptor (PRR)- induced signaling by luciferase reporter assays. HEK293 cells were transfected with an NF-κB luciferase reporter vector, vectors encoding either MyD88, TRAF6, IKKβ, NEMO or p65, and vectors encoding increasing concentrations of PGRN. After 24 h transfection, the overexpressing efficiency of PGRN, MyD88, TRAF6, IKKβ, NEMO or p65 in HEK293 cells was examined by western blotting (Fig 5A). Activation of NF-κB by MyD88 (*p* < 0.01), TRAF6 (*p* < 0.05), IKKβ (*p* < 0.01) and NEMO (*p* < 0.01), but not by p65, was significantly impaired by PGRN (Fig 5A). PGRN did not inhibit p65-mediated NF-κB activation, suggesting that PGRN disrupts the NF-κB pathway immediately upstream of p65, most likely interfering with the IKK complex. To determine whether PGRN also inhibits RIG-I or MAVS-induced IFN-β expression, we co-transfected HEK293 cells with plasmids encoding PGRN and RIG-I, MAVS, TBK1, NEMO or IRF3 and examined the activity of an IFN-β-luc reporter gene. After 24 h transfection, the overexpressing efficiency of PGRN, RIG-I, MAVS, TBK1, NEMO or IRF3 in HEK293 cells was examined by western blotting (Fig 5B). Activation of the IFN-β-luc reporter by RIG-I, MAVS, TBK1, and NEMO was significantly inhibited by PGRN (*p* < 0.01), but not by IRF3 (Fig 5B). These data suggest that PGRN interacts with NEMO to disrupt the NF-κB and IRF3 pathways.

### PGRN binds to NEMO via its Grn CDE domains

**Fig 6:**
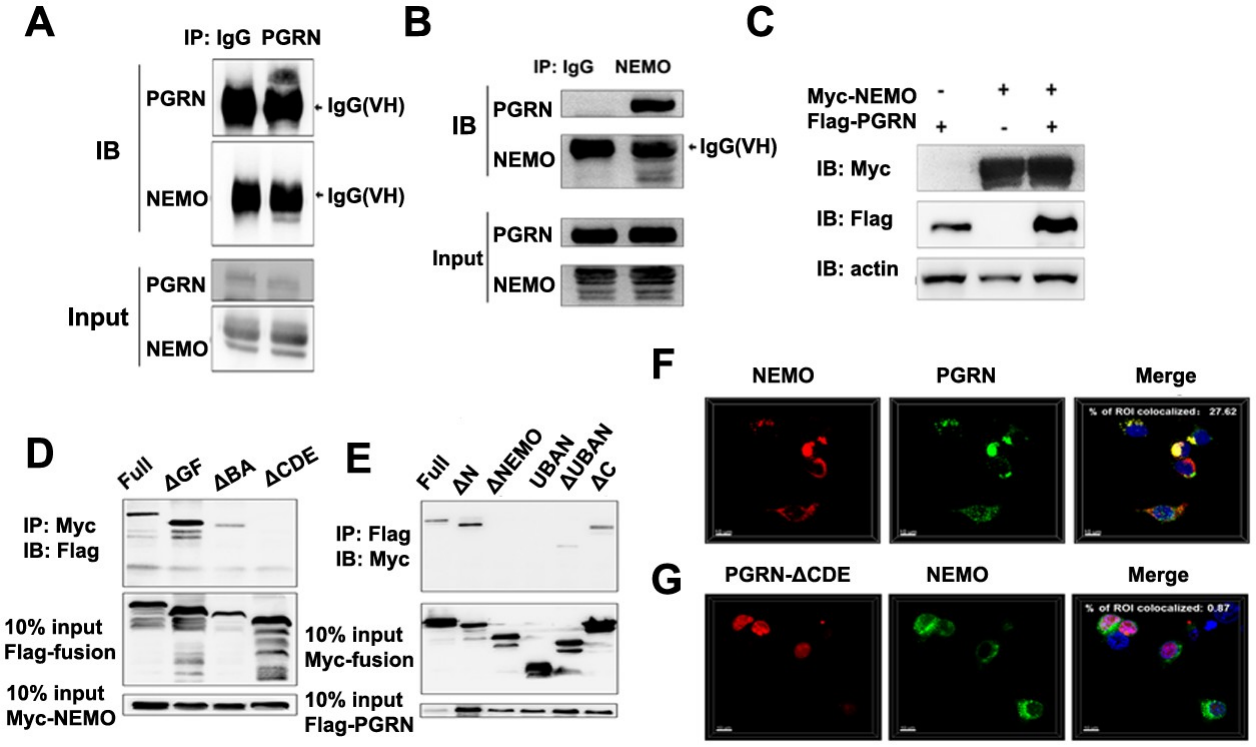
PGRN binds to NEMO. (A) HEK293 cells were infected with PR8 virus at an MOI of 1. Cell lysates were immunoprecipitated with anti-PGRN antibody and probed with anti-NEMO antibody. (B) HEK293 cells were infected with PR8 virus at an MOI of 1. Cell lysates were immunoprecipitated with anti-NEMO antibody and probed with anti-PGRN antibody. (C) HEK293 cells were mock transfected or transfected with vectors encoding FLAG-tagged PGRN for 24 h. Cell lysates were analyzed by immunoblotting with an anti-NEMO antibody. (D) Co-IP analysis of the interaction between Myc-tagged NEMO and FLAG-tagged full-length or mutant PGRN in HEK293 cells. (E) Co-IP analysis of the interaction between FLAG-tagged PGRN and Myc-tagged full-length or mutant NEMO in HEK293 cells. (F) Confocal microscopy of HEK293 cells co-transfected with plasmids encoding FLAG-tagged PGRN and Myc-tagged NEMO and stained with Alexa Fluor 488-conjugated anti-FLAG antibody (green) and Alexa Fluor 555-conjugated anti-Myc antibody (red). The DAPI serves as a marker for nuclei (blue). (G) Confocal microscopy of HEK293 cells co-transfected with vectors encoding FLAG-tagged PGRN mutants and Myc-tagged NEMO. All data are representative of three independent experiments showing similar results.

NEMO is a key adaptor protein in both the NF-κB-mediated proinflammatory signaling pathway and the IRF-mediated type I interferon production pathway. To elucidate the mechanisms of NF-κB and IRF3 activation by PGRN, we examined the endogenous interaction between PGRN and NEMO after PR8 virus infection. The data indicated that endogenous PGRN readily interacted with NEMO (Fig 6A and 6B). Moreover, overexpression of PGRN did not change the level of NEMO in HEK293 cells (Fig 6C).

To determine the binding domains of PGRN involved in interaction with NEMO, we constructed PGRN mutants bearing different deletions (S4A Fig). We also generated a series of NEMO deletion mutants (S4B Fig). Co-IP experiments showed that deletion of the PGRN Grn CDE domains abolished the interaction between PGRN and NEMO, indicating that these PGRN regions were directly involved in NEMO binding (Fig 6D). Furthermore, deletion of residues 91 to 140 in the N-terminal region of NEMO moderately reduced the interaction between NEMO and PGRN, and further deletion of residues 150 to 250 completely abolished the interaction (Fig 6E). The subcellular localization of the interaction between PGRN and NEMO was determined through transfection of HEK293 cells with Myc-tagged NEMO and FLAG-tagged full-length or mutant PGRN lacking the NEMO-binding site. Immunofluorescence studies showed that full-length PGRN colocalized with NEMO in the cytosol (Fig 6F). By contrast, mutant PGRN lacking the Grn CDE domains did not colocalize with NEMO (Fig 6G). Taken together, these findings demonstrate that PGRN binds to NEMO via its Grn CDE domains and colocalizes with NEMO in cytosolic.

### PGRN inhibits the activation of NEMO by reducing K63-linked NEMO ubiquitination

**Fig 7:**
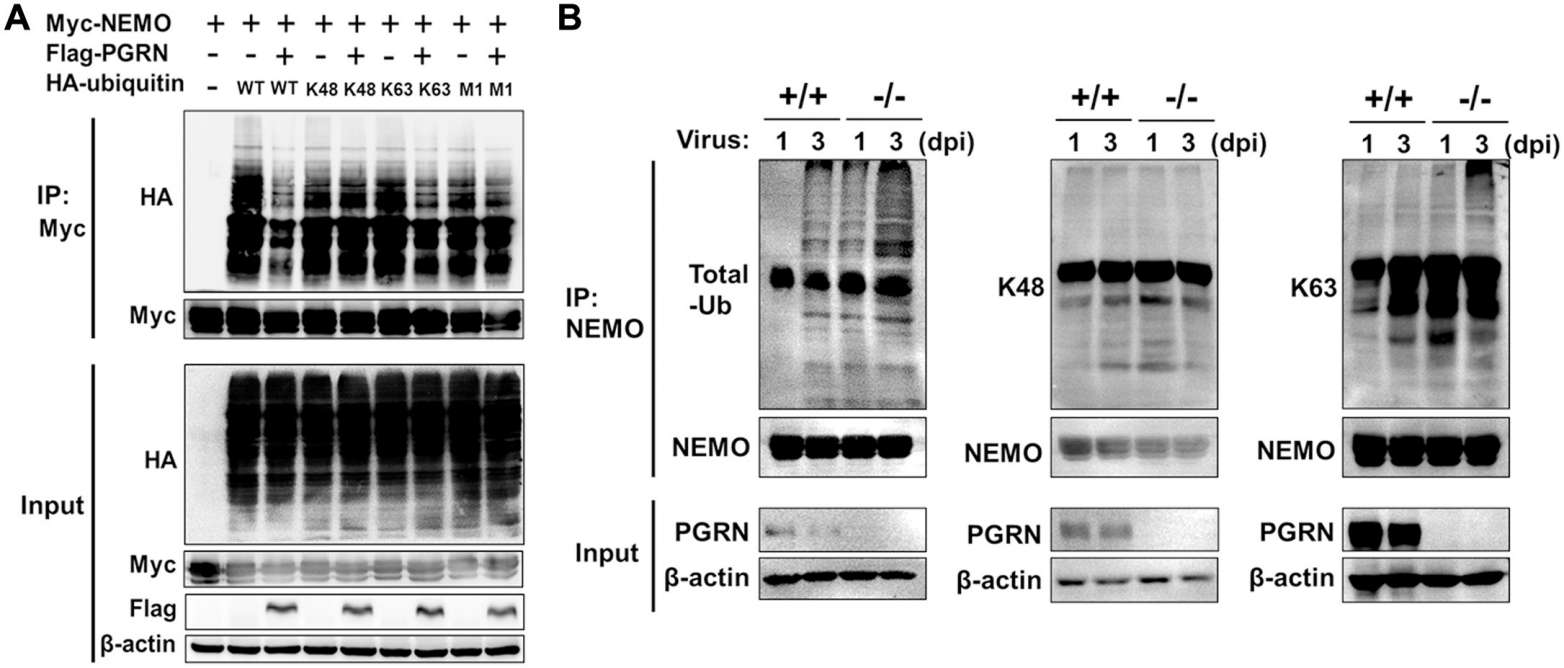
PGRN inhibits K63-linked polyubiquitination of NEMO. (A) Co-IP analysis of NEMO ubiquitination in HEK293 cells transfected with Myc-NEMO, FLAG-PGRN, HA-ubiquitin, HA-ubiquitin-K48, HA-ubiquitin-K63 or HA-ubiquitin-M1. (B) Co-IP analysis of endogenous NEMO ubiquitination in lung tissues from WT and KO mice infected with PR8 virus (1×10^4^ TCID_50_) at 1 and 3 dpi. All data are representative of three independent experiments showing similar results.

NEMO binding to K63-linked polyubiquitin chains is required for the activation of the IKK complex and subsequent signaling. To investigate whether PGRN regulates NEMO polyubiquitination, we transfected HEK293 cells with vectors encoding Myc-tagged NEMO and HA-tagged ubiquitin in the presence or absence of vectors encoding FLAG-tagged PGRN. Overexpression of PGRN remarkably reduced NEMO polyubiquitination (Fig 7A). To further examine PGRN-mediated NEMO polyubiquitination, we transfected HEK293 cells with plasmids encoding Myc-tagged NEMO, FLAG-tagged full-length PGRN, and either HA-ubiquitin, HA-ubiquitin-K48, HA-ubiquitin-K63 or HA-ubiquitin-M1. Co-IP experiments showed that PGRN remarkably reduced NEMO polyubiquitination in the presence of HA-ubiquitin and HA-ubiqutin-K63, but not HA-ubiquitin-K48 and HA-ubiquitin-M1 (Fig 7A). To investigate PGRN-mediated ubiquitination of endogenous NEMO, we examined NEMO polyubiquitination in lung tissues of WT and KO mice after PR8 virus infection. We found that endogenous NEMO was ubiquitinated with K63-linked chains (Fig 7B) and that K63-linked NEMO ubiquitination, but not K48-linked NEMO ubiquitination, was significantly increased in PGRN-deficient lung tissues at 1 and 3 dpi (Fig 7B). These results suggest that PGRN reduces K63-linked NEMO ubiquitination and inhibits the activation of NEMO.

### PGRN recruits A20 to deubiquitinate NEMO

**Fig 8:**
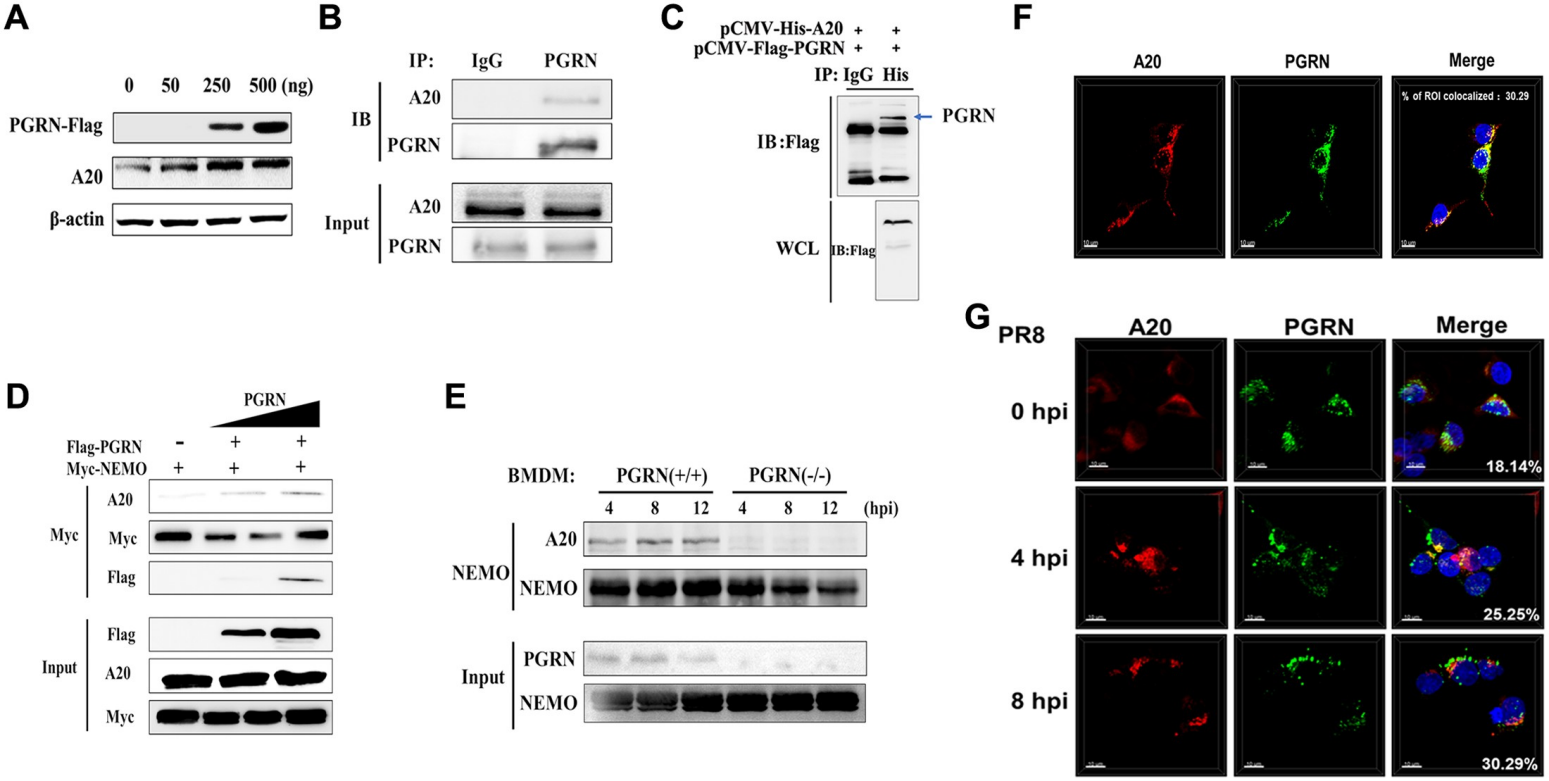
PGRN recruits A20 and facilitates A20-mediated deubiquitination of NEMO. (A) HEK293 cells were mock or transfected with vectors encoding FLAG-tagged PGRN at a concentration of 0, 50, 250, and 500 ng. Cell lysates were analyzed by immunoblotting with an anti-A20 antibody. (B) HEK293 cells were infected with PR8 virus at an MOI of 1. Cell lysates were immunoprecipitated with anti-PGRN antibody and probed with anti-A20 antibody. (C) Immunoblotting of HEK293 cells transfected with plasmids encoding FLAG-tagged PGRN and His-tagged A20 and assayed by Co-IP. (D) HEK293 cells were mock or transfected with vectors encoding FLAG-tagged PGRN and Myc-tagged NEMO and infected with PR8 virus at an MOI of 1 for 8 h. Cell lysates were immunoprecipitated with anti-NEMO antibody to analyze the recruitment of A20 to NEMO. (E) WT and KO BMDMs were infected with PR8 virus at an MOI of 2 for 4, 8 and 12 h. Cell lysates were immunoprecipitated with anti-NEMO antibody to analyze the recruitment of A20 to NEMO. (F) Confocal microscopy of HEK293 cells transfected with plasmids encoding FLAG-tagged PGRN and His-tagged A20. (G) Confocal microscopy of HEK293 cells transfected with plasmids encoding FLAG-tagged PGRN and infected with PR8 virus at an MOI of 1 for 0, 4 and 8 h. All data are representative of three independent experiments showing similar results.

A20 directly interacts with NEMO and blocks the activation of NF-κB by inhibiting K63-linked polyubiquitination of NEMO. Next, we examined whether PGRN affected the interaction between NEMO and A20. PGRN upregulated the expression of A20 in a dose-dependent manner (Fig 8A). Moreover, endogenous PGRN readily interacted with A20 in anti-PGRN immunoprecipitates after influenza virus infection (Fig 8B). Similarly, we found that PGRN interacted with A20 by overexpressing His-tagged A20 and FLAG-tagged full-length PGRN in HEK293 cells. (Fig 8C). Overexpression of PGRN enhanced the level of A20 in anti-NEMO immunoprecipitates after influenza virus infection (Fig 8D). *In vitro* experiments further verified that the level of A20 in anti-NEMO immunoprecipitates from PGRN KO BMDMs was significantly decreased at 4, 8 and 12 hpi (Fig 8E), indicating that PGRN enhances the interaction between A20 and NEMO. Then, we investigated the subcellular localization of the interaction between PGRN and A20 through transfection of HEK293 cells with FLAG-tagged PGRN and His-tagged A20. Immunofluorescence studies indicated that PGRN colocalized with A20 in the cytosol (Fig 8F). Furthermore, influenza virus infection enhanced the interaction between PGRN and A20 (Fig 8G). Collectively, these results imply that PGRN recruits A20 and facilitates the interaction between A20 and NEMO.

**Fig 9:**
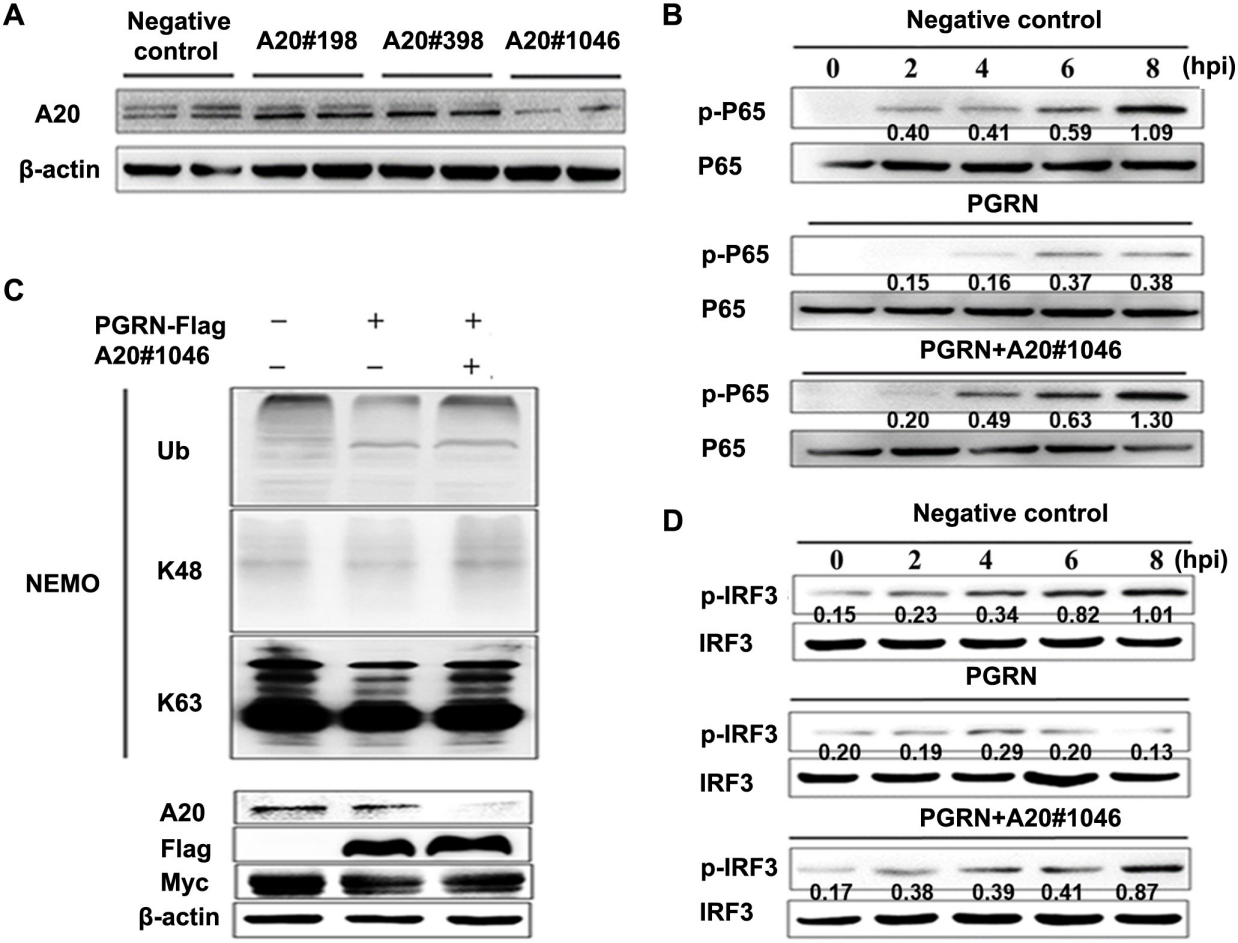
The regulatory effect of PGRN is dependent on A20. (A) Serum-starved HEK293 cells were transfected with negative control or A20-targeting siRNAs (#198, #398 and #1046) to silence A20, and 48 h later, the expression of A20 was measured by western blot. (B) HEK293 cells were co-transfected with a plasmid encoding FLAG-tagged PGRN and either negative control or A20-targeting siRNA #1046, and infected with PR8 virus at an MOI of 1. After 8 hpi, immunoblotting was used to assess K63-linked polyubiquitination of NEMO. (C) HEK293 cells were co-transfected with vectors encoding FLAG-tagged PGRN and either negative control or A20-targeting siRNA #1046, then infected with PR8 virus at an MOI of 1 at the indicated time points. Levels of phosphorylated p65 were measured by western blot. (D) HEK293 cells were co-transfected with vectors encoding FLAG-tagged PGRN and either negative control or A20-targeting siRNA #1046, and infected with PR8 virus at MOI of 1 at the indicated time points. Levels of phosphorylated IRF3 were measured by western blot. All data are representative of three independent experiments showing similar results.

We silenced the expression of A20 in HEK293 cells using A20-targeting siRNAs #198, #398 and #1046, infected the cells with PR8 virus, and analyzed the activation of NF-κB and IRF3. Transfection of HEK293 cells with A20 siRNA#1046 significantly decreased the level of A20 (Fig 9A). PGRN clearly inhibited K63-linked polyubiquitination of NEMO after PR8 virus infection, but this inhibitory effect was decreased in the presence of A20-targeting siRNA (Fig 9B). Furthermore, PGRN inhibited the phosphorylation of NF-κB and IRF3, and silencing of A20 reduced the inhibitory effect of PGRN on NF-κB and IRF3 signaling (Fig 9C and 9D). Therefore, the inhibitory effect of PGRN is largely dependent on A20.

### PGRN inhibits NEMO ubiquitination at Lys264

**Fig 10:**
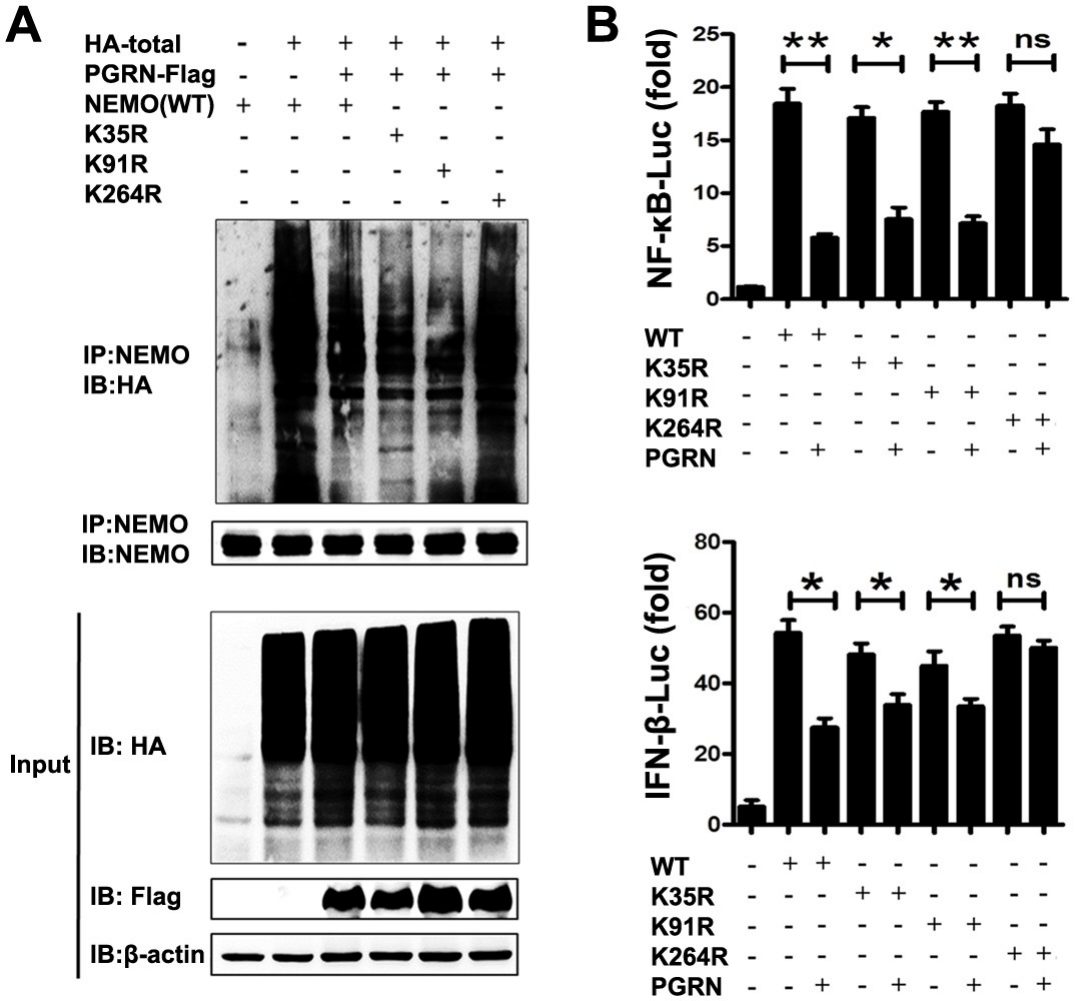
PGRN inhibits NEMO ubiquitination at Lys264. (A) Immunoblotting of HEK293 cells co-transfected with vectors encoding Myc- tagged NEMO or NEMO mutants bearing substitutions of Lys35 (K35R), Lys91 (K91R) or Lys264 (K264R) along with either empty vector or a vector encoding FLAG-tagged PGRN. Data are representative of three independent experiments. (B) HEK293 cells were transfected with (i) empty vector or plasmids encoding NEMO or NEMO mutants bearing substitutions of K35R, K91R or K264R together along with (ii) an empty vector or a PGRN-encoding vector. After 18 h, NF-κB and IFN-β activity was measured using luciferase reporter assays. Data are pooled from three independent experiments performed in triplicate. Error bars indicate SEM. *p < 0.05; **p < 0.01.

We predicted the potential ubiquitination sites on NEMO and found that 22 of 30 lysine residues were potential ubiquitination sites (S2 Table). To determine which lysine residue is the key ubiquitination site in NEMO, we substituted each residue individually with arginine and examined PGRN-induced ubiquitination. The inhibitory effect of PGRN on NEMO polyubiquitination was decreased by the substitution of Lys264 (Fig 10A). To further examine the functionality of the Lys264 site, we showed that overexpression of PGRN inhibited activation of NF-κB and IFN-β reporter gene by wild-type NEMO. However, inhibition of NF-κB and IFN-β activation was not observed for a NEMO mutant bearing a substitution of Lys264 (Fig 10B). Thus, Lys264 might be a key site for PGRN-mediated ubiquitination and function of NEMO.

### Macrophages are the critical source of PGRN during influenza infection

**Fig 11:**
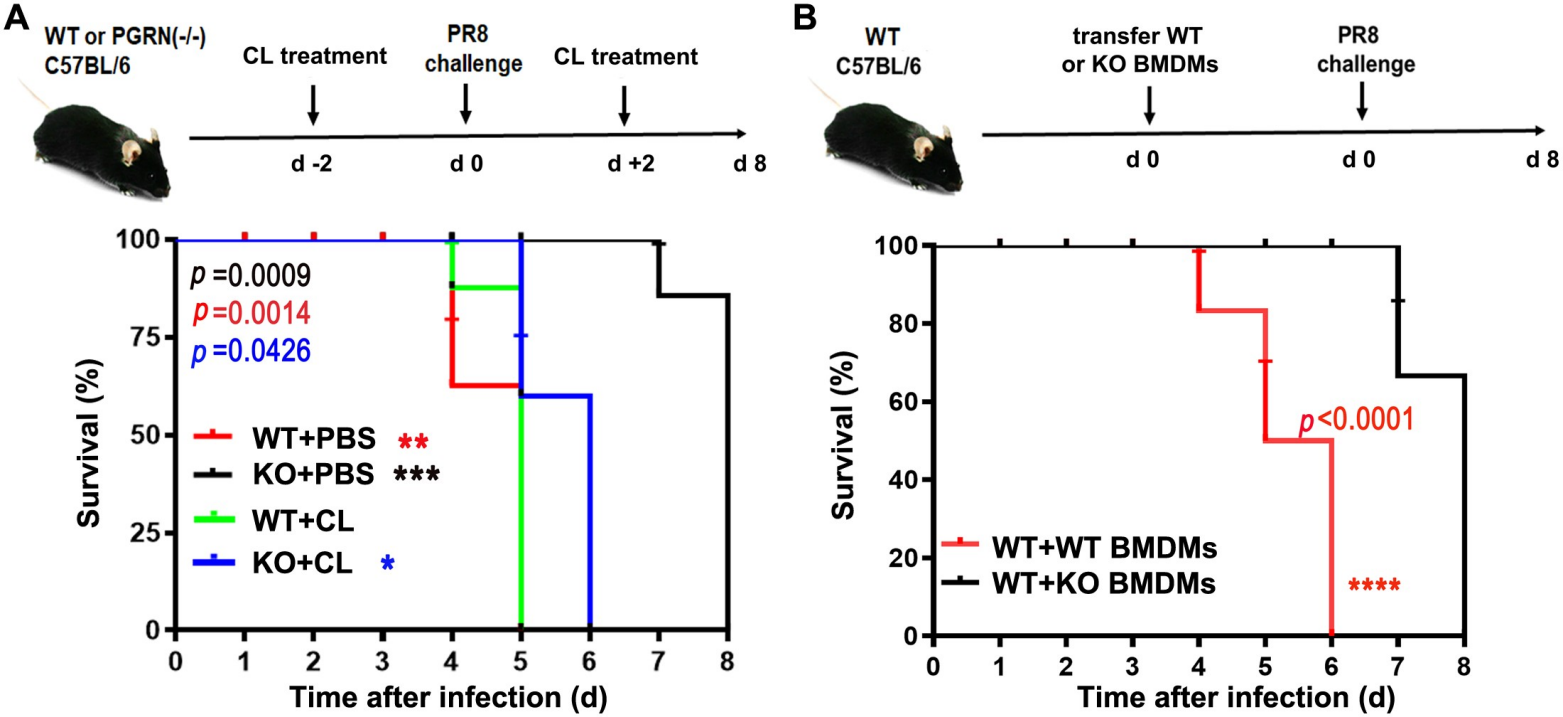
Macrophages are the critical source of PGRN during influenza virus infection. (A) WT and KO mice were treated with 100 μL of CL or PBS containing liposomes two times via the intranasal route, 2 days before infection and at day 2 after infection with PR8 virus at a dose of 1×10^4^ TCID_50_, and the survival rate was measured. Data are pooled from three independent experiments with n = 6 mice per group. By using the log-rank (Mantel-Cox) analysis, the survival of PBS-treated KO mice was significantly different compared to PBS-treated WT mice after PR8 infection (*p* = 0.0014), CL-treatment significantly reduced survival in PR8-infected KO mice compared to PBS-treatment group (*p* = 0.0009), and CL-treatment significantly improved survival in PR8-infected KO mice compared to CL-treatment group (*p* = 0.0426). (B) WT mice were transferred with 3×10^6^ of WT or KO BMDMs by intravenous injection and infected with PR8 virus at a dose of 1×10^4^ TCID_50_, and the survival rate was measured. Data are pooled from three independent experiments with n = 6 mice per group. Kaplan-Meier survival curves are compared using the log-rank (Mantel-Cox) analysis. ****p < 0.0001.

Alveolar macrophages are critical for protection from respiratory failure and associated morbidity after influenza virus infection[22]. Furthermore, some evidence suggests that macrophage-secreted PGRN plays a central role in host defense[21, 23], inflammatory response[24, 25] and tumor growth[26]. Clodronate Liposomes (CL) is taken up by phagocytic macrophage (Mφ) and accumulates in the cytosol, resulting in Mφ death and depletion[27]. Administration of CL has been widely used to selectively deplete Mφ in mouse models[28, 29]. Our data also demonstrated that CL treatment significantly reduced the number of lung macrophages (CD11b+CD11c-Ly6G-) in mice (*p* < 0.01), but did not change the percentage of lung dendritic cells (CD11b-CD11c+Ly6G-) (*p* > 0.05) (S5 Fig). To evaluate the contribution of macrophage-derived PGRN during influenza virus infection, we treated WT and KO mice with 100 μL of CL or PBS containing liposomes two times via the intranasal route, 2 days before PR8 infection and at day 2 after PR8 infection, and measured the survival rate. Our findings revealed that PBS-treated WT and KO mice were dead on 5 and 8 dpi respectively, whereas the CL-treated WT and KO mice died on day 5 and 6 after PR8 virus infection, suggesting that depletion of PGRN-deficient AMs exacerbates influenza virus infection in KO mice (Fig 11A). To further explore the function of WT and PGRN-deficient macrophages during influenza infection, we adoptively transferred WT or KO BMDMs into WT recipients by intravenous injection and measured the survival rate after PR8 infection. We found that transfer of WT or KO BMDMs significantly increased the lung macrophage numbers compared to PBS-treated mice (*p* < 0.05), but there was no significant difference between WT and KO BMDMs recipients (*p* > 0.05) (S6A Fig). In addition, the Ki-67 expression was similar in lung macrophages from PBS, WT and KO BMDMs recipients (*p* > 0.05) (S6B Fig). As predicted, we found that WT BMDMs recipients had a higher PGRN production in serum (*p* < 0.01) and in BALF (*p* < 0.05) than KO BMDMs recipients (S7 Fig). In addition, we found that WT mice adoptively transferred with KO BMDMs were dead on 8 dpi, whereas control mice died on 6 dpi (Fig 11B). Collectively, macrophages play a critical source of PGRN during influenza virus infection, and PGRN-deficient macrophages transfer delay the mortality caused by influenza virus infection in mice.

### Therapeutic studies of PGRN antibodies in mice

**Fig 12:**
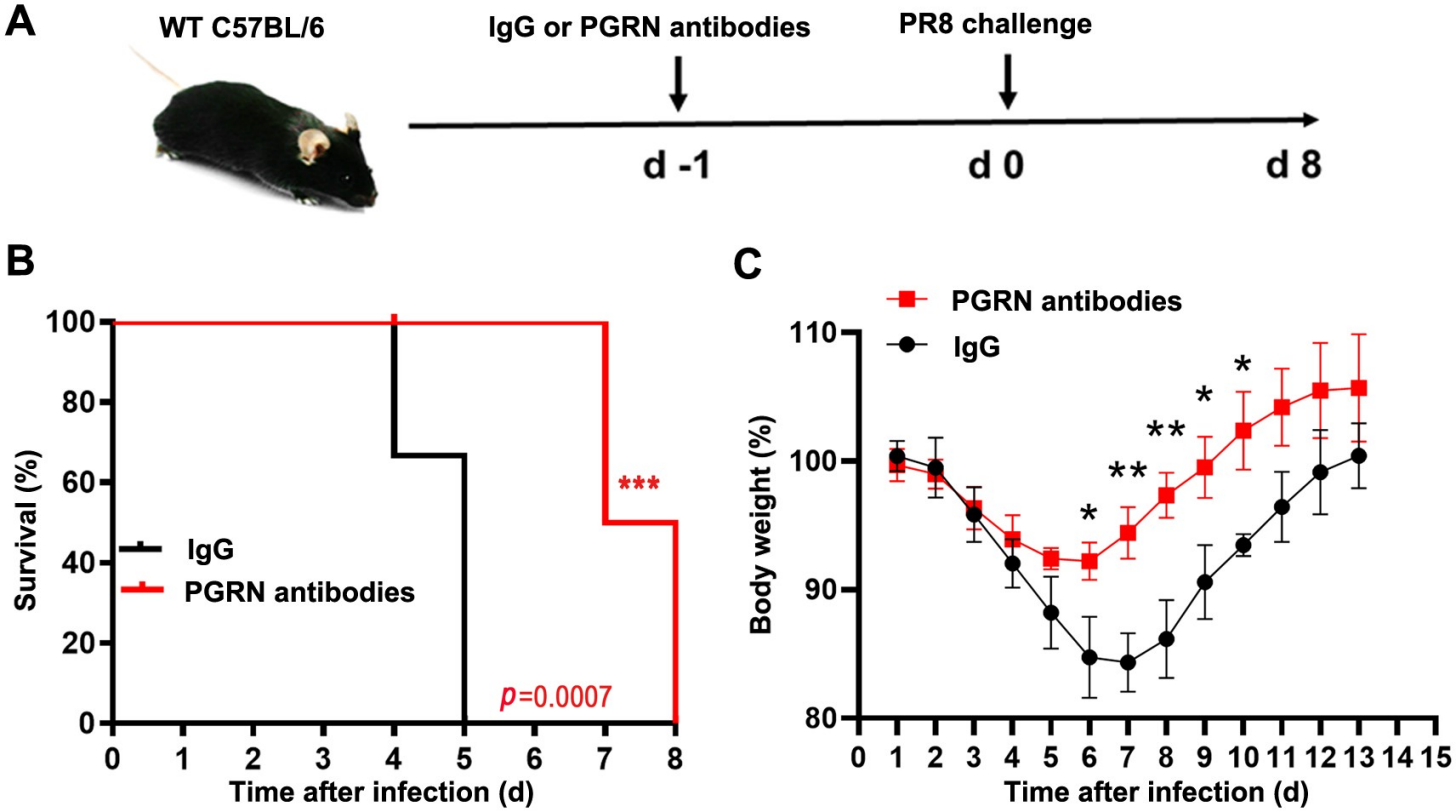
PGRN antibodies protect against lethal influenza virus infection in mice. (A) Six-week-old male C57BL/6 mice were passively administered 200 μg of IgG control or PGRN polyclonal antibodies via intraperitoneal injection 1 day prior to inoculation with PR8 virus at a lethal dose of 1×10^4^ TCID_50_ via the intranasal route. (B) The survival of two group mice was monitored daily. Data are pooled from three independent experiments with n = 10 mice per group. Kaplan-Meier survival curves are compared using the log-rank (Mantel-Cox) analysis. ***p<0.001. (C) Six-week-old male C57BL/6 mice were passively administered 200 μg of IgG control or PGRN polyclonal antibodies via intraperitoneal injection 1 day prior to inoculation with PR8 virus at a dose of 50 TCID_50_ via the intranasal route. Changes in body weight were monitored daily. Each data point represents as the means ± SEMs and is representative of three independent experiments. *p < 0.05, **p < 0.01.

To further evaluate whether PGRN neutralization could protect against influenza virus-induced lethality *in vivo*, we treated 6-week-old male C57BL/6 mice with 200 μg of IgG control or PGRN polyclonal antibodies via intraperitoneal injection at 1 day prior to inoculation with PR8 virus at a lethal dose of 1×10^4^ TCID_50_ (Fig 12A). The results revealed that mice treated with IgG control died by 5 dpi (Fig 12B). In contrast, mice treated with PGRN polyclonal antibodies died by 7 dpi (Fig 12B). The mice treated with PGRN polyclonal antibodies began to regain weight on day 6, 7, 8, 9 and 10 after infection with PR8 virus at a dose of 50 TCID_50_ (Fig 12C). These data suggest that PGRN antibodies show a therapeutic effect on mortality caused by influenza virus lethal infection in mice.

## Discussion

Viral infection triggers a series of signaling cascades that result in expression of type I IFNs, which play key roles in cellular antiviral responses. Identifying the regulators of innate antiviral responses that control type I IFNs is helpful for understanding and manipulating of antiviral responses. In the present study, we found that avian and human influenza virus-induced PGRN negatively regulated the production of type I IFNs by inhibiting activation of NF-κB and IRF3 signaling. Furthermore, PGRN directly bound to NEMO and recruited A20 to deubiquitinate K63-linked polyubiquitin chains on NEMO at Lys264, leading to the suppression of NF-κB and IRF3 activation. Our findings demonstrate a novel function of PGRN in influenza A virus infection.

We found that PGRN was strongly upregulated by virus infection in H7N9- infected human patients and mouse models, and PGRN-deficient mice were resistant to avian and human influenza virus infection, suggesting that PGRN plays a key role in influenza virus infection. Our data are consistent with other published results. For example, Brandes etal. show that H1N1 infection induces PGRN expression in the lungs of mice[15], and Luo et al. demonstrate that PGRN is elevated in serum samples from H1N1-infected patients and PGRN KO mice are resistant to H1N1 virus infection[30]. Since there are currently no clinical biomarkers to predict fatal outcomes of lethal virus infection, further studies are needed to evaluate whether PGRN is strongly linked with disease severity and mortality in influenza virus-infected animals and patients.

Upon influenza virus infection, viral RNA can be sensed by various pattern recognition receptors (PRRs) including Toll-like receptors (TLRs), NOD-like receptors (NLRs) and RIG-I-like receptors (RLRs), resulting in the activation of various transcription factors, especially NF-κB and IRF3. NF-κB and IRF3 collaborate to induce type I IFNs, including IFN-α and IFN-β, which are a central event in the innate immune response[31, 32]. In our study, we found that overexpression of PGRN inhibited influenza virus-induced phosphorylation of p65 and IRF3, and KO mice showed stronger activation of p65 and IRF3 than WT mice after virus infection. The inhibitory effect of PGRN on NF-κB activation is consistent with a recent study, which reveals that PGRN deficiency leads to excessive NF-κB activation[33]. IFN-α and -β receptor subunit 1 (IFNAR1)-deficient mice are resistant to *Listeria monocytogenes* infection[34–36], and a very recent report shows that expression of IFNAR1 significantly differs between *Grn*^-/-^, *Grn*^+/-^ and *Grn*^+/+^ mice[37]. These findings suggest that type I IFN expression is regulated by PGRN. In the present study, we found that overexpression of PGRN inhibited influenza virus-induced production of type I IFNs through the activation of the NF-κB and IRF3 pathways, supporting a prominent role of PGRN in the inhibition of type I IFN signaling. Influenza viruses use multiple strategies to evade host immune defense. For example, the influenza A virus-encoded NS1 protein plays a major role in preventing the activation of NF-κB and inhibiting type I IFN-mediated antiviral effects[38]. The negative regulation of type I IFNs by PGRN demonstrated here represents a novel type I IFN evasion mechanism of influenza A virus.

TLR activation leads to the recruitment of adaptor molecules, such as MyD88 and TRIF, which act on a series of downstream signaling molecules. These molecules synthesize K63-linked polyubiquitin chains on themselves and other proteins, which then recruit the IKK complex as well as the kinases TBK1/IKKε through binding to NEMO[39, 40]. By contrast, activation of RIG-I and MDA-5 by influenza virus results in the recruitment of the MAVS protein[40–43]. The IKKα/IKKβ/NEMO complex is essential for influenza virus-induced activation of NF-κB. However, activation of IRF3 is regulated by the TBK1/IKKε/NEMO complex[32, 44, 45], which suggests that NEMO is a critical adaptor in both the NF-κB and IRF3 signaling pathways. Published reports have provided strong evidence that K63 polyubiquitination plays a critical role in signal transduction through multiple pathways, including those triggered by TLRs, RLRs and NLRs[46]. For example, post-translational modification of NEMO by ubiquitination plays a key role in regulating its function in the IKK complex[22]. Our findings uncover a role of PGRN as a regulator of innate immune responses.

Deubiquitination is the reverse process of ubiquitination and is mediated by a group of proteins called deubiquitinating enzymes (DUBs)[47]. The activation of IKK is negatively regulated by DUBs that cleave K63 ubiquitin chains, such as A20, CYLD and Otulin[48–50]. A20 is an important negative regulator of innate immune responses[51], and harbors a deubiquitination enzyme domain. A20 restricts cellular activation signals by cleaving activating K63-linked polyubiquitin chains from target signaling proteins[52–55], including NEMO. We found that PGRN was involved in removal of K63-linked ubiquitin to NEMO through induction and recruitment of the deubiquitinase A20. Accumulating evidence supports PGRN’s anti-inflammatory role in various disease conditions[10, 11, 56–59], but its exact mechanisms need to be better elucidated. Our findings suggest that PGRN recruits A20, which providing a natural brake on inflammation[60], and blocks the production of type I IFNs and proinflammatory cytokines. This represents a novel mechanism of the anti-inflammatory function of PGRN.

After influenza virus infection, viral single-stranded RNA is detected by RIG-I, resulting in TRAF3 and TRAF6-dependent transcription of type I IFNs. Our finding does not rule out the possibility that other E3 enzymes may also be involved in polyubiquitination of NEMO in a concerted manner, such as the TRIM family of ubiquitin E3 ligases.

**Fig 13:**
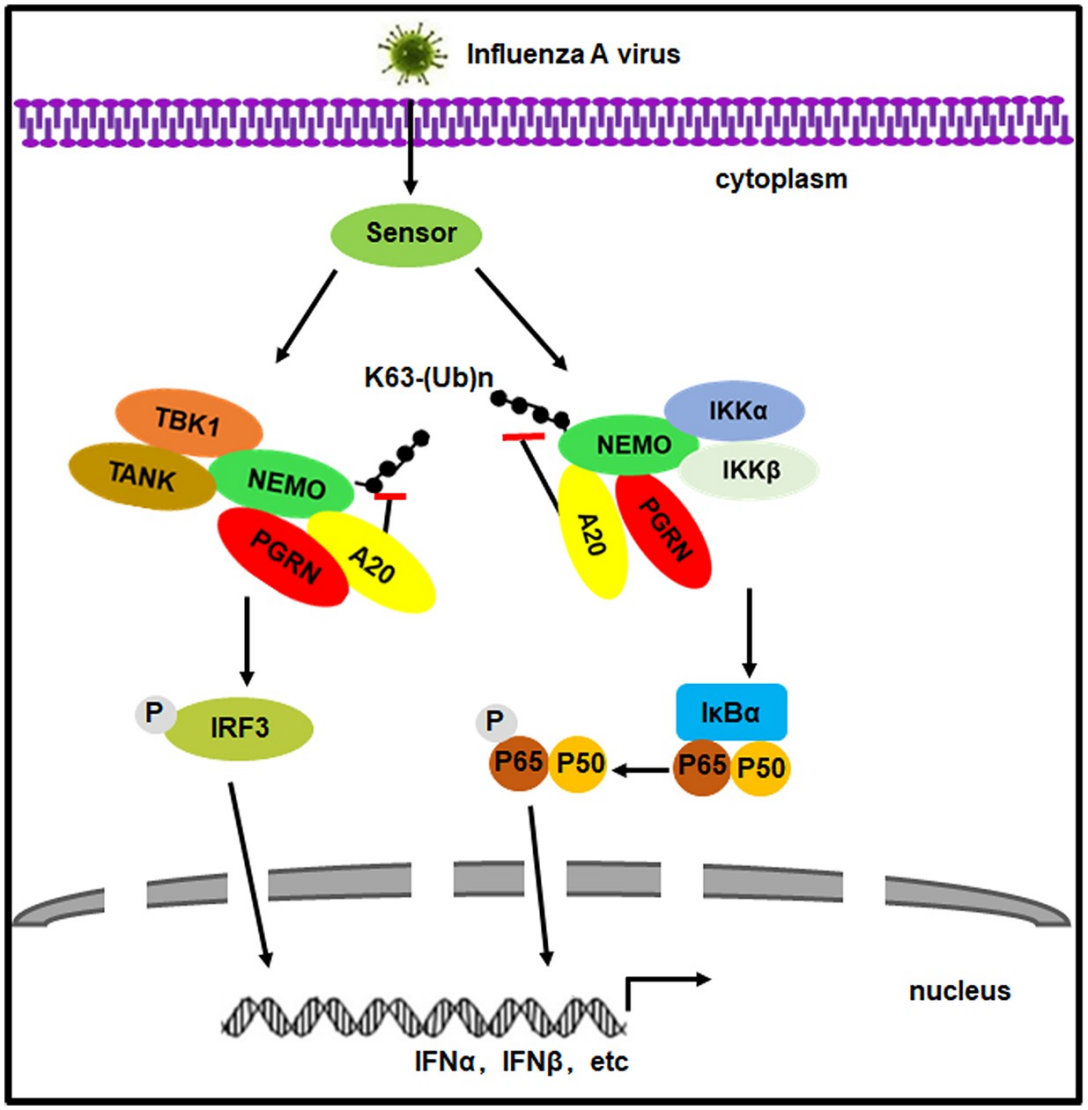
Proposed model of the roles of PGRN in inhibition of type I IFN signaling upon influenza virus infection. In brief, influenza virus induces the upregulation of PGRN to negatively regulate NF-κB and IRF3 activation, thereby suppressing type I IFN responses.

Collectively, our study shows that PGRN induced by influenza virus negatively regulates type I IFN production by inhibiting the activation of NF-κB and IRF3 signaling through the deubiquitination of NEMO and recruitment of A20 (a proposed model illustrated in Fig 13). This study highlights a type I interferon evasion mechanism in influenza A virus infection. These findings also help us understand the physiological role and crosstalk of PGRN in antiviral innate immunity.

## Supporting information

S1 Fig PGRN level is elevated during influenza virus infection *in vivo*. (A) PGRN levels in the BALF of mice challenged with PR8 virus at a dose of 1×10^2^ TCID_50_ at the indicated time points were determined by ELISA. Data are representative of three independent experiments performed in triplicate. *p<0.05, **p<0.01. (B) PGRN levels in the sera of mice challenge with PR8 virus at a dose of 1×10^2^ TCID_50_ at the indicated time points were determined by ELISA. Data are representative of three independent experiments performed in triplicate. *p<0.05, **p<0.01. (C) PGRN levels in the sera of healthy volunteers (n = 6) and H7N9 virus-infected patients (n = 6) were measured using ELISA. Data are representative of three independent experiments performed in triplicate. Error bars indicate SEM. ***p<0.001. (D) WT mice (n = 3 per group) were infected with H5N1 (1×10^2^ TCID_50_), PR8 (1×10^2^ TCID_50_) or H9N2 (1×10^3^ TCID_50_) viruses. PGRN expression in lung tissue sections from mock-infected and PR8-infected mice was examined by immunohistochemistry. Representative sections of one mouse out of three are shown. (E) PGRN mRNA expression in A549 cells infected by UV-irradiated H1N1 (uvH1N1) and H9N2 (uvH9N2) viruses at an MOI of 1 were compared with live virus infection at 6 hpi. Data are representative of three independent experiments. Error bars indicate SEM. ***p<0.001. (F) PGRN expression in HEK293 cells 48 h after transfection with indicating PR8 virus FLAG-tagged protein-coding pRK5 plasmids. β-actin is shown as a loading control. NC represents negative control, and EV represents empty vector. Data are representative of three independent experiments.
(TIF)

S2 Fig The genotyping results of WT and PGRN KO mice. The genomic DNA was extracted and purified from mouse tail samples using the DNeasy Blood and Tissue Kit (Qiagen). The PCR was performed to identify the wild type (468 bp) and mutant mice (211 bp) using primers provided by the Jackson Laboratory.
(TIF)

S3 Fig PGRN decreases the subcellular translocation of p65 and IRF3 after PR8 virus infection. (A) Serum-starved HEK293 cells were transfected with control or PGRN-encoding plasmids. 48 h after transfection, cells were infected with PR8 virus at an MOI of 2, and the subcellular localizations of p65 were assessed. (B) Serum-starved HEK293 cells were transfected with control or PGRN-encoding plasmids. 48 h after transfection, cells were infected with PR8 virus at an MOI of 2, and the subcellular localization of IRF3 was assessed. All data are representative of three independent experiments showing similar results.
(TIF)

S4 Fig Schematic diagram of full-length PGRN, NEMO and truncated mutants. (A) Schematic diagram of full-length PGRN and truncation mutants. (B) Schematic diagram of full-length NEMO and truncation mutants.
(TIF)

S5 Fig CL treatment reduces the number of lung macrophages in mice. WT mice were treated with 100 μL of CL or PBS containing liposomes two times via the intranasal route, once every 2 days, and the single cell lung suspensions was prepared and stained with CD11b-APC-cy7, CD11c-PE and Ly6G-FITC antibodies. The number of lung macrophages and dendritic cells was analyzed by FACS. Data are representative of three independent experiments with n = 3 mice per group. *p<0.05, **p<0.01.
(TIF)

S6 Fig The number and survival of lung macrophages between WT BMDMs recipients and KO BMDMs recipients is similar. (A) WT mice were transferred with PBS control, 3×10^6^ of WT or KO BMDMs by intravenous injection and the lung macrophage numbers were evaluated at day 3 post-injection by FACS. Data are representative of three independent experiments with n = 3 mice per group. *p<0.05, **p<0.01. (B) WT mice were transferred with PBS control, 3×10^6^ of WT or KO BMDMs by intravenous injection and the lung macrophage were sorted by FACS at day 3 post-injection. The Ki-67 expression in lung macrophages from PBS control, WT BMDMs recipients and KO BMDMs recipients was measured by western blot. Data are representative of three independent experiments. Each lane represents one mouse sample.
(TIF)

S7 Fig WT BMDMs recipients have a higher PGRN production in serum and BALF than KO BMDMs recipients. WT mice were transferred with cell culture medium control, 3×10^6^ of WT or KO BMDMs by intravenous injection and PGRN levels in serum and BALF were measured at day 3 post-injection. Data are representative of three independent experiments with n = 3 mice per group. *p<0.05, **p<0.01.
(TIF)

S1 Table List of primer pairs used for real-time PCR in this study. (DOCX)

S2 Table Potential ubiquitination sites within the NEMO molecule. The potential for ubiquitination of NEMO lysine residues was predicted from its primary amino acid sequence using “UbPred: predictor of protein ubiquitination sites”.
(DOCX)
