# Correction: Induction of PGRN by influenza virus inhibits the antiviral immune responses through downregulation of type I interferons signaling

**DOI:** 10.1371/journal.ppat.1008321

**Published:** 2020-02-03

**Authors:** Fanhua Wei, Zhimin Jiang, Honglei Sun, Juan Pu, Yipeng Sun, Mingyang Wang, Qi Tong, Yuhai Bi, Xiaojing Ma, George Fu Gao, Jinhua Liu

There is an error in Fig 8. In Fig 8D, the Myc panel was edited incorrectly during the composition of the final figure. The authors confirm that these changes do not alter their findings. The authors have provided raw, uncropped blots as Supporting Information. Please see the correct Fig 8 here.

**Fig 8:**
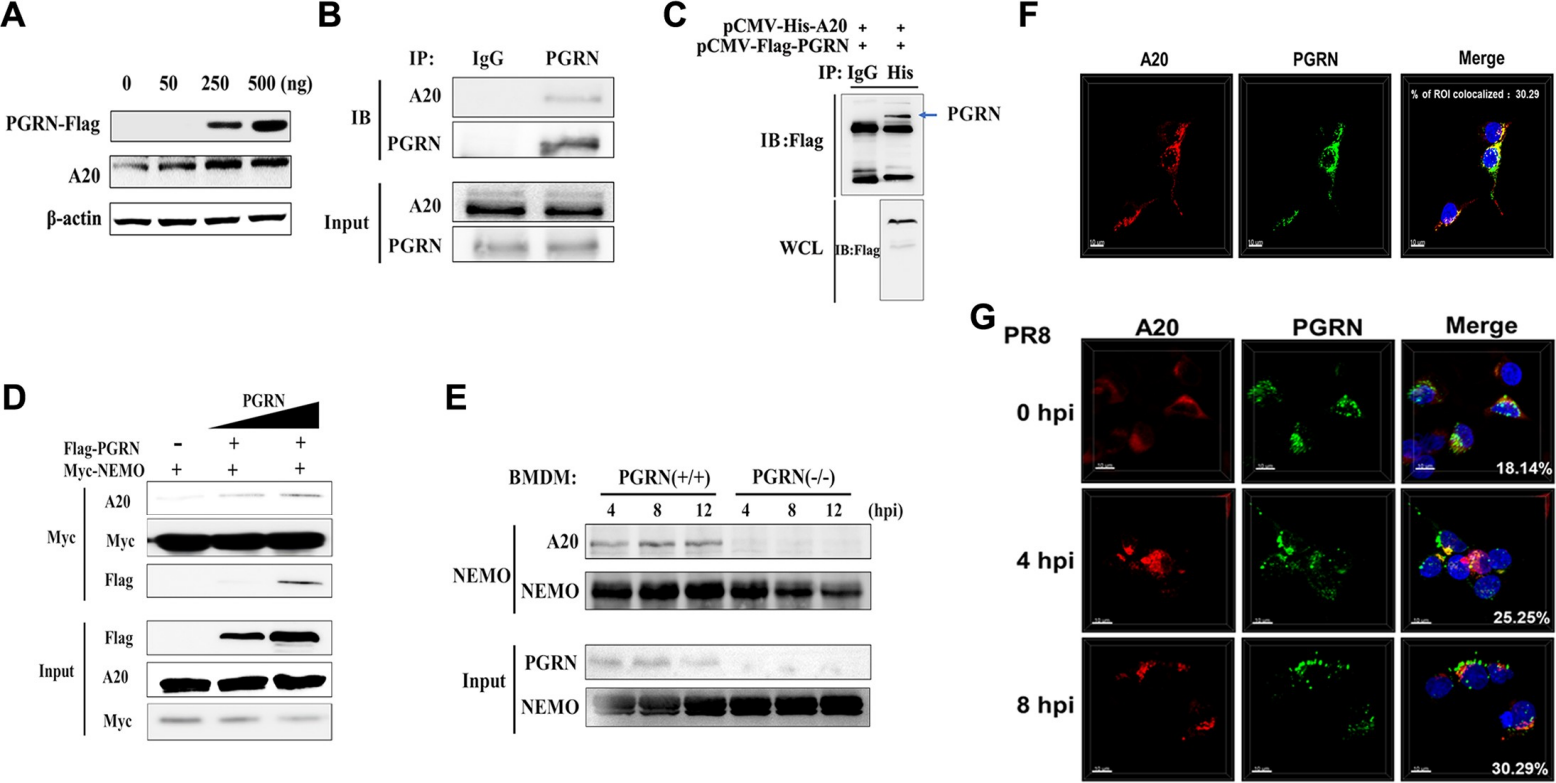
PGRN recruits A20 and facilitates A20-mediated deubiquitination of NEMO. (A) HEK293 cells were mock or transfected with vectors encoding FLAG-tagged PGRN at a concentration of 0, 50, 250, and 500 ng. Cell lysates were analyzed by immunoblotting with an anti-A20 antibody. (B) HEK293 cells were infected with PR8 virus at an MOI of 1. Cell lysates were immunoprecipitated with anti-PGRN antibody and probed with anti-A20 antibody. (C) Immunoblotting of HEK293 cells transfected with plasmids encoding FLAG-tagged PGRN and His-tagged A20 and assayed by Co-IP. (D) HEK293 cells were mock or transfected with vectors encoding FLAG-tagged PGRN and Myc-tagged NEMO and infected with PR8 virus at an MOI of 1 for 8 h. Cell lysates were immunoprecipitated with anti-NEMO antibody to analyze the recruitment of A20 to NEMO. (E) WT and KO BMDMs were infected with PR8 virus at an MOI of 2 for 4, 8 and 12 h. Cell lysates were immunoprecipitated with anti-NEMO antibody to analyze the recruitment of A20 to NEMO. (F) Confocal microscopy of HEK293 cells transfected with plasmids encoding FLAG-tagged PGRN and His-tagged A20. (G) Confocal microscopy of HEK293 cells transfected with plasmids encoding FLAG-tagged PGRN and infected with PR8 virus at an MOI of 1 for 0, 4 and 8 h. All data are representative of three independent experiments showing similar results.

## Supporting information

S1 Fig Original data with raw uncropped blots. (DOCX)

## References

[1] Wei F, Jiang Z, Sun H, Pu J, Sun Y, Wang M, et al (2019) Induction of PGRN by influenza virus inhibits the antiviral immune responses through downregulation of type I interferons signaling. PLoS Pathog 15(10): e1008062 10.1371/journal.ppat.1008062 31585000 PMC6795447

