# Supplementary figures and images for: Induction of PGRN by influenza virus inhibits the antiviral immune responses through downregulation of type I interferons signaling
Source: PLoS Pathog. 2019 Oct 4;15(10):e1008062. doi: 10.1371/journal.ppat.1008062 (PMC6795447; doi:10.1371/journal.ppat.1008062)

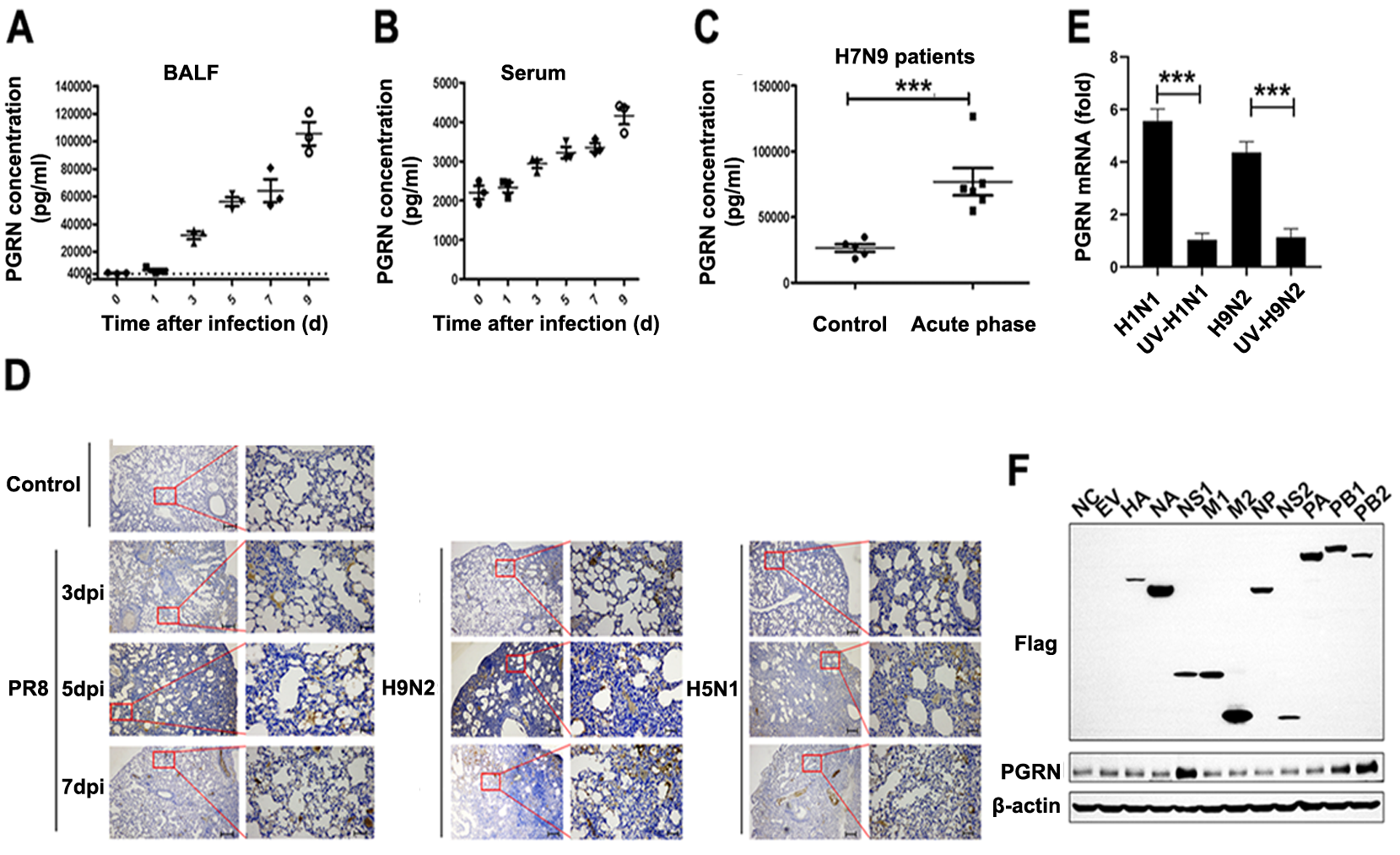

Supplement: S1 Fig — (A) PGRN levels in the BALF of mice challenged with PR8 virus at a dose of 1×10^2^ TCID50 at the indicated time points were determined by ELISA. Data are representative of three independent experiments performed in triplicate. *p<0.05, **p<0.01. (B) PGRN levels in the sera of mice challenge with PR8 virus at a dose of 1×10^2^ TCID50 at the indicated time points were determined by ELISA. Data are representative of three independent experiments performed in triplicate. *p<0.05, **p<0.01. (C) PGRN levels in the sera of healthy volunteers (n = 6) and H7N9 virus-infected patients (n = 6) were measured using ELISA. Data are representative of three independent experiments performed in triplicate. Error bars indicate SEM. ***p<0.001. (D) WT mice (n = 3 per group) were infected with H5N1 (1×10^2^ TCID50), PR8 (1×10^2^ TCID50) or H9N2 (1×10^3^ TCID50) viruses. PGRN expression in lung tissue sections from mock-infected and PR8-infected mice was examined by immunohistochemistry. Representative sections of one mouse out of three are shown. (E) PGRN mRNA expression in A549 cells infected by UV-irradiated H1N1 (uvH1N1) and H9N2 (uvH9N2) viruses at an MOI of 1 were compared with live virus infection at 6 hpi. Data are representative of three independent experiments. Error bars indicate SEM. ***p<0.001. (F) PGRN expression in HEK293 cells 48 h after transfection with indicating PR8 virus FLAG-tagged protein-coding pRK5 plasmids. β-actin is shown as a loading control. NC represents negative control, and EV represents empty vector. Data are representative of three independent experiments. (TIF) [file ppat.1008062.s001.tif]

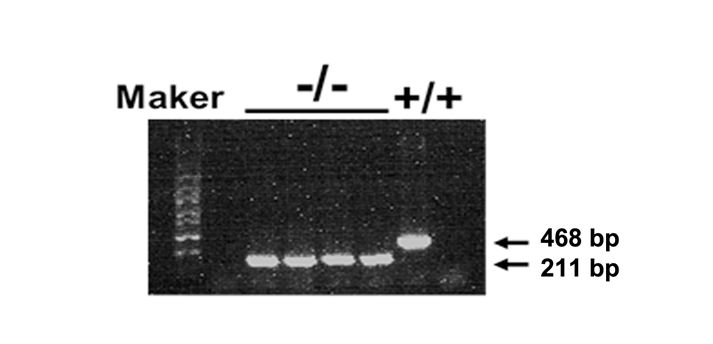

Supplement: S2 Fig — The genomic DNA was extracted and purified from mouse tail samples using the DNeasy Blood and Tissue Kit (Qiagen). The PCR was performed to identify the wild type (468 bp) and mutant mice (211 bp) using primers provided by the Jackson Laboratory. (TIF) [file ppat.1008062.s002.tif]

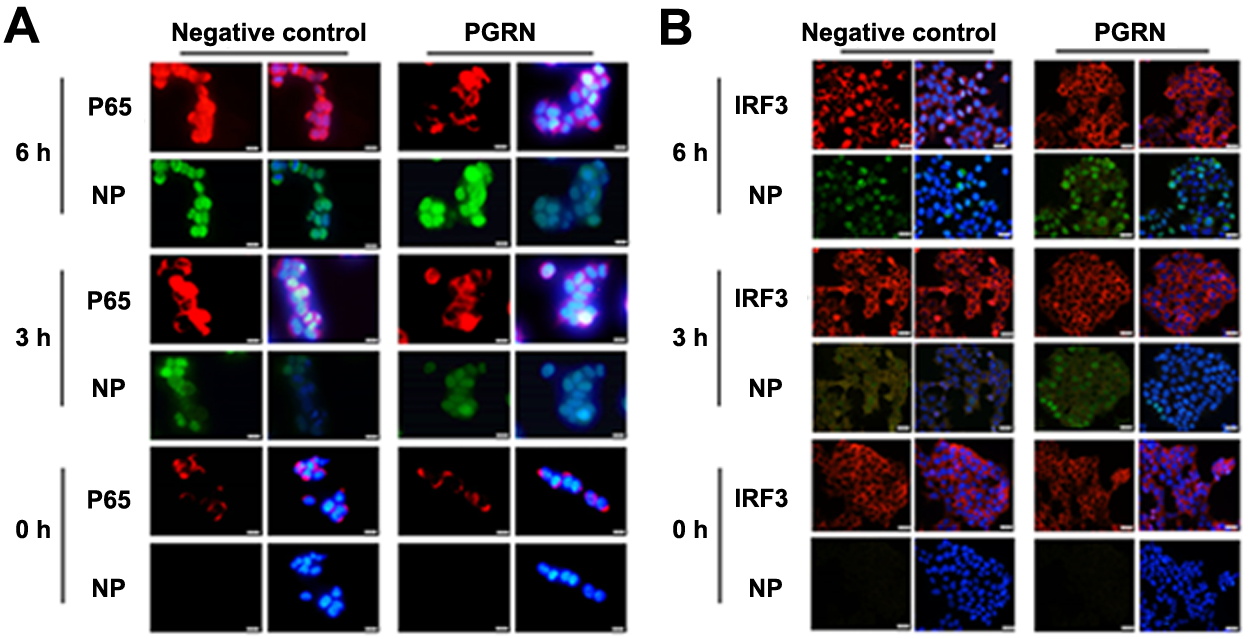

Supplement: S3 Fig — (A) Serum-starved HEK293 cells were transfected with control or PGRN-encoding plasmids. 48 h after transfection, cells were infected with PR8 virus at an MOI of 2, and the subcellular localizations of p65 were assessed. (B) Serum-starved HEK293 cells were transfected with control or PGRN-encoding plasmids. 48 h after transfection, cells were infected with PR8 virus at an MOI of 2, and the subcellular localization of IRF3 was assessed. All data are representative of three independent experiments showing similar results. (TIF) [file ppat.1008062.s003.tif]

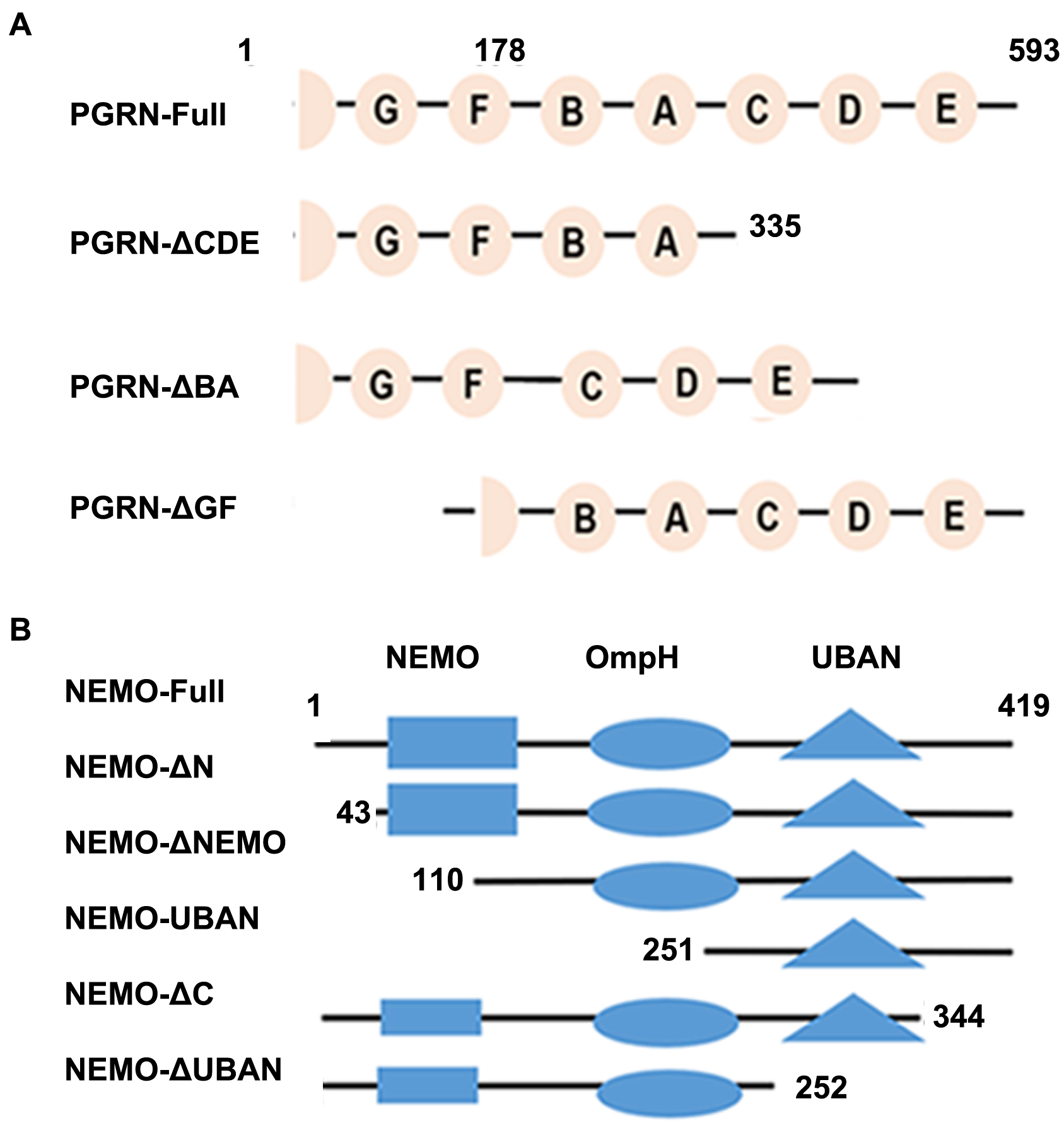

Supplement: S4 Fig — (A) Schematic diagram of full-length PGRN and truncation mutants. (B) Schematic diagram of full-length NEMO and truncation mutants. (TIF) [file ppat.1008062.s004.tif]

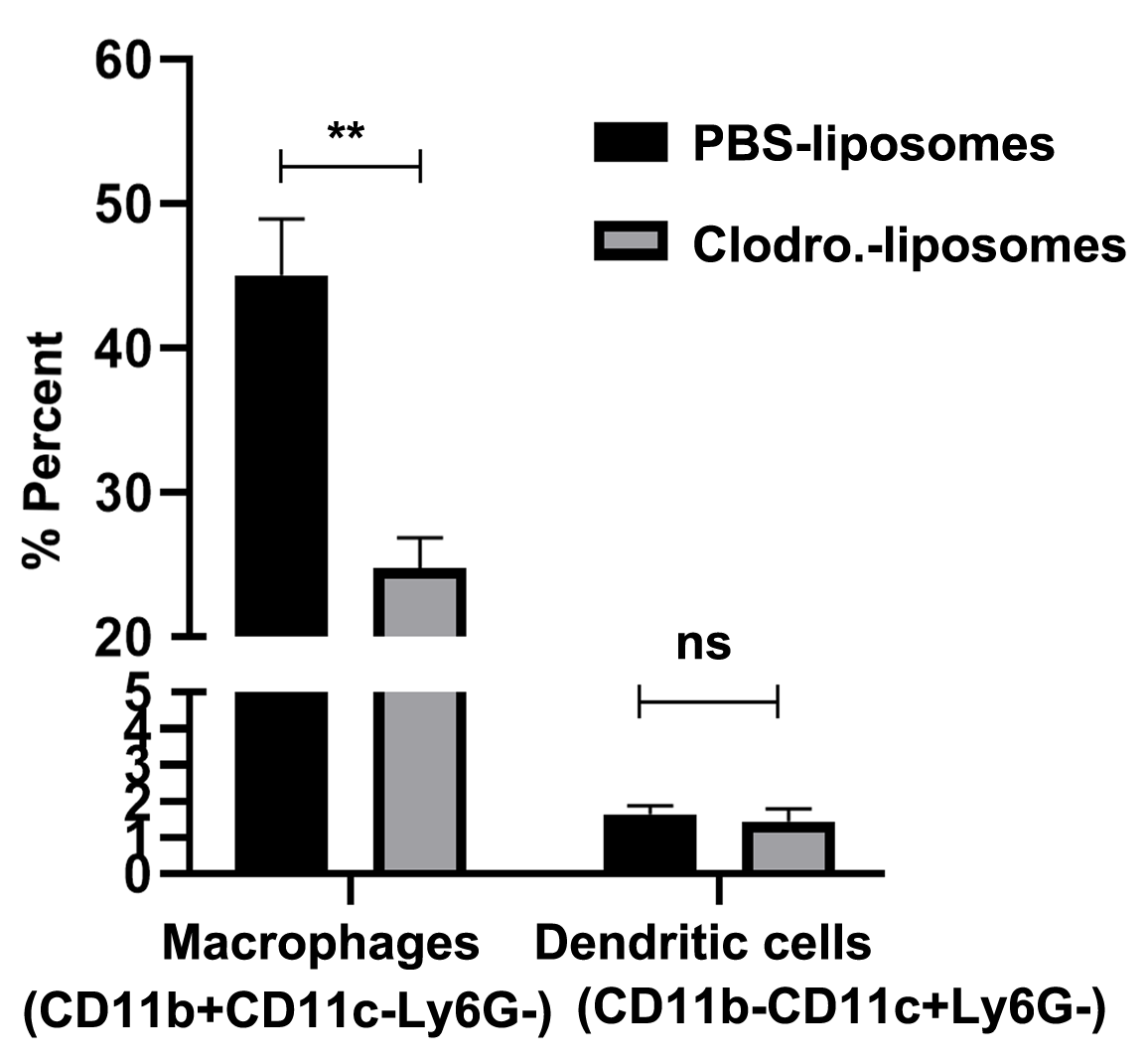

Supplement: S5 Fig — WT mice were treated with 100 μL of CL or PBS containing liposomes two times via the intranasal route, once every 2 days, and the single cell lung suspensions was prepared and stained with CD11b-APC-cy7, CD11c-PE and Ly6G-FITC antibodies. The number of lung macrophages and dendritic cells was analyzed by FACS. Data are representative of three independent experiments with n = 3 mice per group. *p<0.05, **p<0.01. (TIF) [file ppat.1008062.s005.tif]

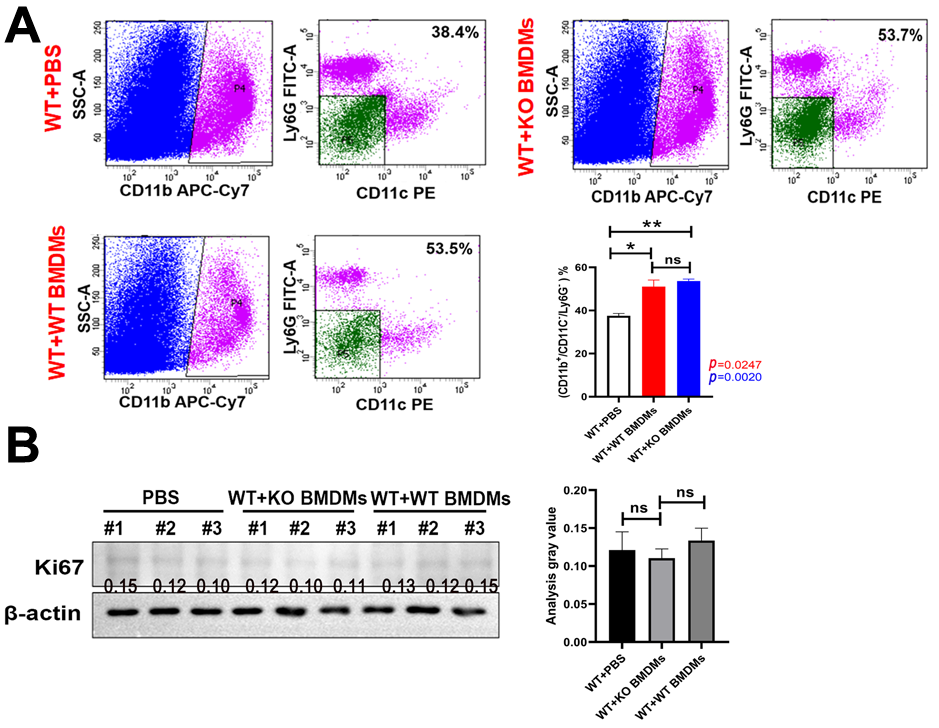

Supplement: S6 Fig — (A) WT mice were transferred with PBS control, 3×10^6^ of WT or KO BMDMs by intravenous injection and the lung macrophage numbers were evaluated at day 3 post-injection by FACS. Data are representative of three independent experiments with n = 3 mice per group. *p<0.05, **p<0.01. (B) WT mice were transferred with PBS control, 3×10^6^ of WT or KO BMDMs by intravenous injection and the lung macrophage were sorted by FACS at day 3 post-injection. The Ki-67 expression in lung macrophages from PBS control, WT BMDMs recipients and KO BMDMs recipients was measured by western blot. Data are representative of three independent experiments. Each lane represents one mouse sample. (TIF) [file ppat.1008062.s006.tif]

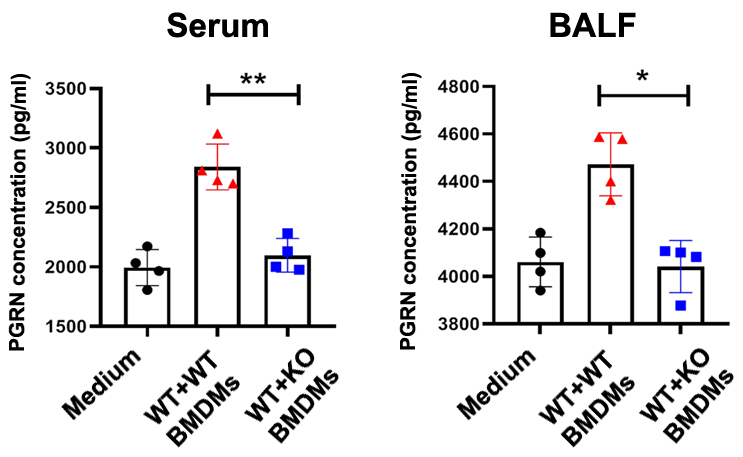

Supplement: S7 Fig — WT mice were transferred with cell culture medium control, 3×10^6^ of WT or KO BMDMs by intravenous injection and PGRN levels in serum and BALF were measured at day 3 post-injection. Data are representative of three independent experiments with n = 3 mice per group. *p<0.05, **p<0.01. (TIF) [file ppat.1008062.s007.tif]
