# Supplementary material for: Induction of PGRN by influenza virus inhibits the antiviral immune responses through downregulation of type I interferons signaling
Source: PLoS Pathog. 2019 Oct 4;15(10):e1008062. doi: 10.1371/journal.ppat.1008062 (PMC6795447; doi:10.1371/journal.ppat.1008062)
Supplement: S1 Table — (DOCX) [file ppat.1008062.s008.docx]

**Supplementary Table 1. List of primer pairs used for real-time PCR in this study.**

| Primers | Forward sequence | Reverse sequence |
| --- | --- | --- |
| IFN-β | 5′-AGACTATTGTTGTACGTCTCC-3′ | 5′-CAGTAATAGCTCTTCAAGTGG-3′ |
| ISG-15 | 5′-CTCTGAGCATCCTGGTGAGGAA-3′ | 5′-AAGGTCAGCCAGAACAGGTCGT-3′ |
| viperin | 5′-TGCTGGCTGAGAATAGCATTAGG-3′ | 5′-GCTGAGTGCTGTTCCCATCT-3′ |
| CCL-5 | 5′-CAGCAGCAAGTGCTCCAATCTT-3′ | 5′-TTCTTGAACCCACTTCTTCTCTGG-3′ |
| GAPDH | 5′-AACTTTGGCATTGTGGAAGG-3′ | 5′-ACACATTGGGGGTAGGAACA-3′ |
