# Supplementary material for: Induction of PGRN by influenza virus inhibits the antiviral immune responses through downregulation of type I interferons signaling
Source: PLoS Pathog. 2019 Oct 4;15(10):e1008062. doi: 10.1371/journal.ppat.1008062 (PMC6795447; doi:10.1371/journal.ppat.1008062)
Supplement: S2 Table — The potential for ubiquitination of NEMO lysine residues was predicted from its primary amino acid sequence using “UbPred: predictor of protein ubiquitination sites”. (DOCX) [file ppat.1008062.s009.docx]

**NEMO Ubiquitination Sites**


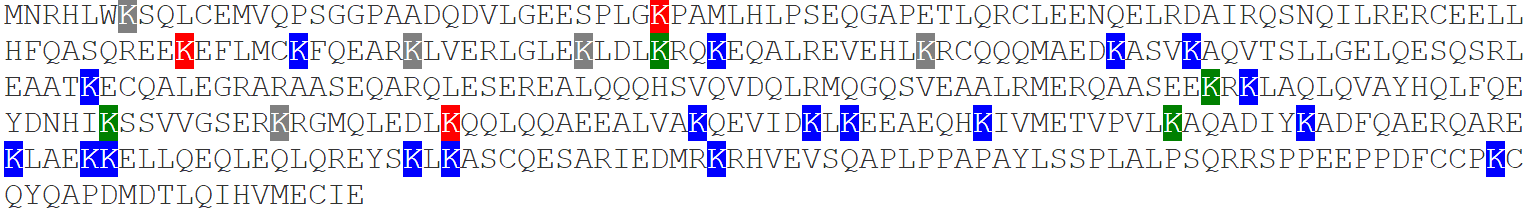


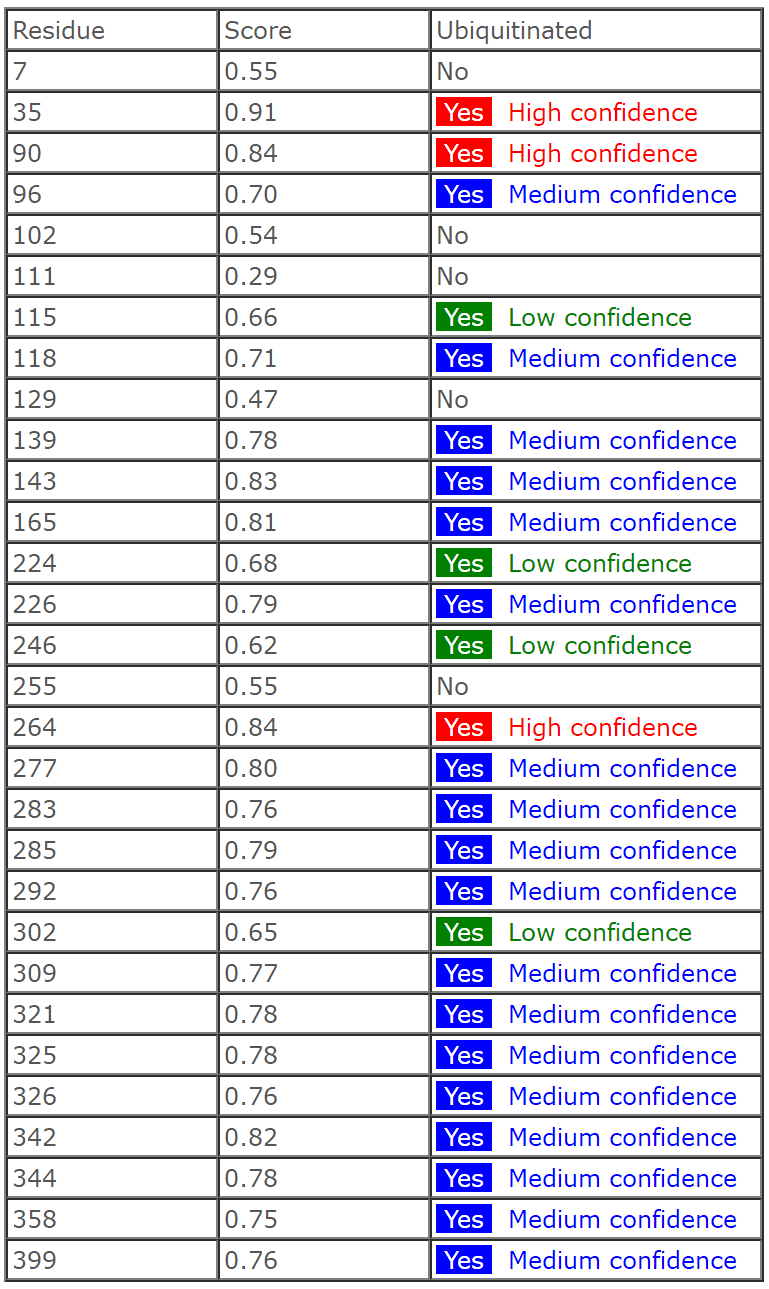


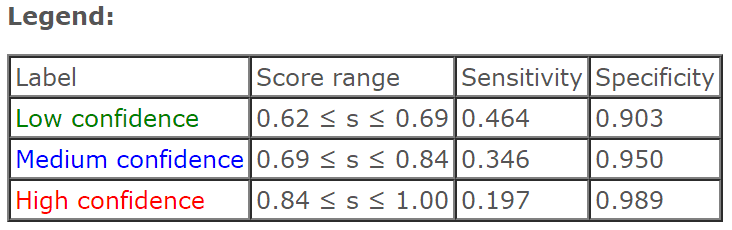


**Supplementary Table 2. Potential ubiquitination sites within the NEMO molecule.**

The potential for ubiquitination of NEMO lysine residues was predicted from its primary amino acid sequence using “UbPred: predictor of protein ubiquitination sites”.
