# Supplementary figures and images for: Correction: Induction of PGRN by influenza virus inhibits the antiviral immune responses through downregulation of type I interferons signaling
Source: PLoS Pathog. 2020 Feb 3;16(2):e1008321. doi: 10.1371/journal.ppat.1008321 (PMC6996799; doi:10.1371/journal.ppat.1008321)

**
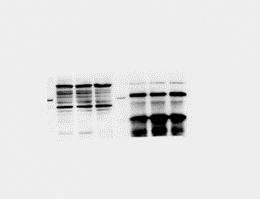

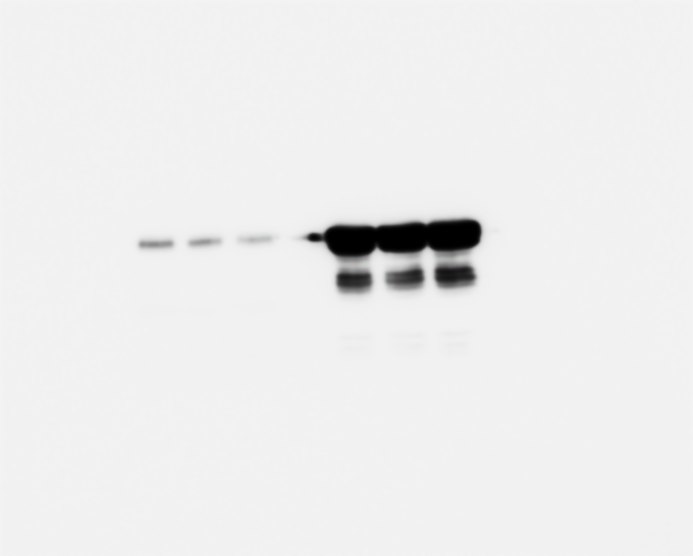
Myc**

**
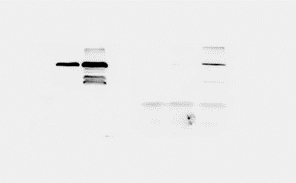
A20**

**Flag**

Supplement: S1 Fig — (DOCX) [file ppat.1008321.s001.docx]
